# Identification of glycogene-based prognostic signature as a potential biomarker and therapeutic target in sarcopenia

**DOI:** 10.1097/JS9.0000000000000833

**Published:** 2023-10-12

**Authors:** Dong Xu Chen, Yi Da Wang

**Affiliations:** a Department of Anesthesiology, West China Second Hospital of Sichuan University, Sichuan Province; b Key Laboratory of Birth Defects and Related Diseases of Women and Children, Sichuan University, Ministry of Education; c Key Laboratory of Bio-Resource and Eco-Environment of Ministry of Education, College of Life Science, Sichuan University, Chengdu, People’s Republic of China

*Dear Editor,*

Sarcopenia is an age-related skeletal muscle disorder characterized by a syndrome of decreased muscle mass, strength, and physical functioning and the resulting adverse consequences such as decreased physical performance and quality of life. According to a recent article published in the *International Journal of Surgery*, Beumer *et al*.^1^ conducted a multicenter retrospective cohort study to examine the impact of sarcopenia on overall survival. The study findings revealed that the effect of sarcopenia on overall survival varies depending on the region, with a significant association observed in the East. Specifically, in Japan, a strong association between sarcopenia and overall survival was observed [hazard ratio (HR) 2.00, 95% confidence interval (CI) 1.230–3.08, *P*=0.002]. However, this association was not found in the Netherlands (HR 0.76, 95% CI 0.42–1.36, *P*=0.351). Importantly, the interaction term demonstrated that this regional difference was statistically significant (HR 0.37, 95% CI 0.19–0.73, *P*=0.005). It remains unclear whether regional differences in the impact of sarcopenia on patient outcomes can be explained by gene-based factors. In recent years, a variety of potential biomarkers have been proposed based on studies of the pathogenesis of sarcopenia, but there are currently no validated biomarker criteria. The ‘glycogenes’, or genes related to glycosylation, account for 1% of the human genome and include genes coding glycosyl-transferases, glycosidases, and transporters for nucleotide sugars. Glycosylation is a common post-translational modification, and the glycogenes involved play a crucial role in many human diseases, including diabetes, cardiovascular diseases, and neurodegenerative diseases. Therefore, the main objective of this study is to assess the correlation between glycogenes and sarcopenia and to deepen the mechanistic understanding of the role of glycogenes in physiology or pathology^2^.

The present data were obtained from the GEO (Gene Expression Omnibus) database GSE167186. In this data, we performed transcriptome profiling on lower limb muscle biopsies from 72 young, old, and sarcopenic subjects using bulk RNA-seq (*N*=72) and single-nuclei RNA-seq (*N*=17). We use 40 old and sarcopenic subjects in the following analysis. One hundred eighty-five genes based on previously reported statistics^3^ were inserted into the search tool STRING (https://string-db.org/) for retrieving interacting genes, and the network was constructed by selecting only the two types of sources of interactions, experimental and database, as well as by selecting 100 interactions units in the setup interface (eTable 1, Supplemental Digital Content 1, http://links.lww.com/JS9/B187). The KEGG (Kyoto Encyclopedia of Genes and Genomes) enrichment analysis (realized by the R package clusterProfiler) was then performed on all protein genes except the glycogenes, and the results were taken as adjust-*P* less than 0.01. The results of the KEGG enrichment analysis are summarized in the following Figure 1. A three-level network diagram of glycogene-target gene-pathway was formed (Fig. 1D). By using cytoHubba (http://apps.cytoscape.org/apps/cytohubba), the center gene of the corresponding PPIs (Protein–Protein Interactions) network was indicated (eTable 2, Supplemental Digital Content 1, http://links.lww.com/JS9/B187)^4^.

**Figure 1:**
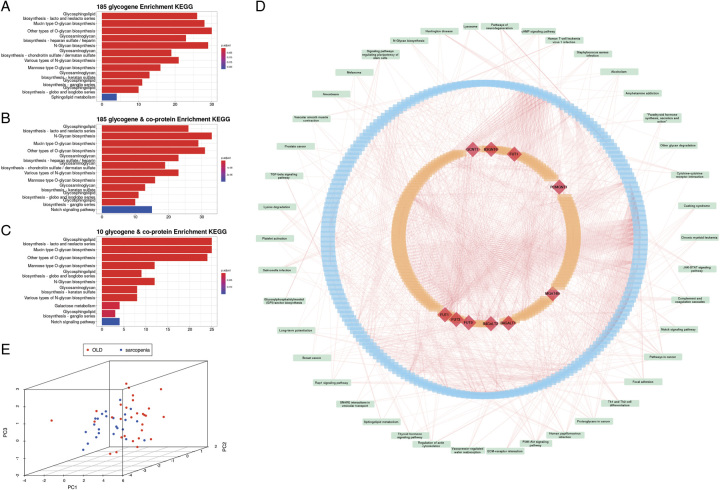
KEGG () enrichment analysis and protein-pathway interaction network. (A) KEGG enrichment results of 185 glycogenes. (B) KEGG enrichment results of 185 glycogenes with target genes. (C) KEGG enrichment results of 10 key glycogenes with target genes. (D) Glycogenes-target protein-pathway interaction network, with 185 glycogenes indicated in orange, target proteins interacting with glycogenes in blue, and pathways associated with glycogenes or target genes in green; the inner circle in red indicates the 10 key glycogenes screened by cytoHubba. (E) PCA, principal component analysis. KEGG, Kyoto Encyclopedia of Genes and Genomes.

We got 10 glycogenes: B3GNT6, B4GALT1, B4GALT2, FUT1, FUT3, FUT9, GALNT3, GCNT1, MGAT4B, and POMGNT1. The center glycogenes enrichment results with target genes indicated that they are mainly involved in the biosynthesis of various types of *O*-glycan, *N*-glycan, and sphingolipids (Fig. 1A–C), which are bioprocesses that tend to take place in the mitochondria as well, and its abnormality leads to the blockage of the energy metabolism of the mitochondria^5^. In addition, the initial steps of *N*-glycosylation guide protein folding and its quality control. *O*-glycosylation stabilizes these domains after folding, allowing them to be secreted. On the cell surface, glycoconjugates form glycocalyx, which provides a barrier and protective layer that protects the plasma membrane from physical stress and shapes the cell surface. Later, PCA, principal component analysis, based on the glycogenes revealed that these glycogenes distinguish the elderly healthy population from the sarcopenia population (Fig. 1E).

Our results suggest that glycogenes can be used as potential molecular markers for screening sarcopenia, providing a basis for scientific and rational evaluation of patients’ sarcopenia status and subsequent rational interventions. A comprehensive understanding of mitochondrial pathophysiology in sarcopenia necessitates distinguishing between the effects of intrinsic muscular aging and those of lifestyle habits and common age-associated conditions. To truly comprehend sarcopenia at an individual level, it is crucial to recognize that the phenotypic variability of this condition may be the result of a mix of factors operating at various levels. These factors can range from the extramuscular environment to specific mitochondrial processes occurring in skeletal myocytes. Therefore, future advancements in the field call for the incorporation of new analytical tools to obtain a comprehensive characterization of the sarcopenic patient. However, whether the association between glycogenes and sarcopenia is region-dependent remains further study.

## Ethical approval

GEO belongs to public databases. The patients involved in the database have obtained ethical approval. Users can download relevant data for free for research and publish relevant articles. Our study is based on open-source data, so there are no ethical issues or other conflicts of interest.

## Sources of funding

None.

## Author contribution

D.X.C. and Y.D.W.: study concept, design, and acquisition of data; Y.D.W.: data cleaning and analysis, and interpreted the data; D.X.C.: drafting the manuscript. All the authors approved the final manuscript as submitted and agree to be accountable for all aspects of the work.

## Conflicts of interest disclosure

The authors declare no conflicts of interest.

## Research registration unique identifying number (UIN)

GEO belongs to public databases. The patients involved in the database have obtained ethical approval. Users can download relevant data for free for research and publish relevant articles. Our study is based on open-source data, so there is no additional registration required.

## Guarantor

Both authors.

## Data availability statement

The data presented in this study are available on request from the corresponding author.

## Supplementary Material

SUPPLEMENTARY MATERIAL

## References

[1] Beumer BR Takagi K Buettner S . Impact of sarcopenia on clinical outcomes for patients with resected hepatocellular carcinoma: a retrospective comparison of Eastern and Western cohorts. Int J Surg 2023;109:2258–2266. 37204461 10.1097/JS9.0000000000000458 PMC10442104

[2] Chao Z Yu-Hang Z De-Xing L . Advance of signal transduction pathways in normal cell. Ibrain 2016;2:20–25.

[3] Narimatsu H . Construction of a human glycogene library and comprehensive functional analysis. Glycoconj J 2004;21:17–24. 15467393 10.1023/B:GLYC.0000043742.99482.01

[4] Altaf-Ul-Amin M Shinbo Y Mihara K . Development and implementation of an algorithm for detection of protein complexes in large interaction networks. BMC Bioinform 2006;7:207. 10.1186/1471-2105-7-207 PMC1473204 16613608

[5] Lunde IG Aronsen JM Kvaløy H . Cardiac O-GlcNAc signaling is increased in hypertrophy and heart failure. Physiol Genomics 2012;44:162–172. 22128088 10.1152/physiolgenomics.00016.2011

