# Supplementary material for: Identification of glycogene-based prognostic signature as a potential biomarker and therapeutic target in sarcopenia
Source: Int J Surg. 2023 Oct 12;110(1):630–2. doi: 10.1097/JS9.0000000000000833 (PMC10793808; doi:10.1097/JS9.0000000000000833)
Supplement: SUPPLEMENTARY MATERIAL [file js9-110-630-s001.docx]

**Appendix**

eTable 1. Glycogene-target gene interaction information.

| Note 1 | Note 2 | Node1_string_id | Node2_string_id | Neighborhood_on_chromosome | Gene_fusion | Phylogenetic_cooccurrence | Homology | Coexpression | Experimentally_determined_interaction | Database_annotated | Automated_textmining | Combined_score |
| --- | --- | --- | --- | --- | --- | --- | --- | --- | --- | --- | --- | --- |
| B3GNT6 | ST6GALNAC1 | 9606.ENSP00000484640 | 9606.ENSP00000156626 | 0 | 0 | 0 | 0 | 0 | 0 | 0.9 | 0 | 0.9 |
| B3GNT6 | GALNT18 | 9606.ENSP00000484640 | 9606.ENSP00000227756 | 0 | 0 | 0 | 0 | 0 | 0 | 0.65 | 0 | 0.65 |
| B3GNT6 | GALNT8 | 9606.ENSP00000484640 | 9606.ENSP00000252318 | 0 | 0 | 0 | 0 | 0 | 0 | 0.65 | 0 | 0.65 |
| B3GNT6 | GALNT5 | 9606.ENSP00000484640 | 9606.ENSP00000259056 | 0 | 0 | 0 | 0 | 0 | 0 | 0.65 | 0 | 0.65 |
| B3GNT6 | GALNT7 | 9606.ENSP00000484640 | 9606.ENSP00000265000 | 0 | 0 | 0 | 0 | 0 | 0 | 0.65 | 0 | 0.65 |
| B3GNT6 | GALNT1 | 9606.ENSP00000484640 | 9606.ENSP00000269195 | 0 | 0 | 0 | 0 | 0 | 0 | 0.65 | 0 | 0.65 |
| B3GNT6 | GALNT10 | 9606.ENSP00000484640 | 9606.ENSP00000297107 | 0 | 0 | 0 | 0 | 0 | 0 | 0.65 | 0 | 0.65 |
| B3GNT6 | C1GALT1C1 | 9606.ENSP00000484640 | 9606.ENSP00000304364 | 0 | 0 | 0 | 0 | 0 | 0 | 0.9 | 0 | 0.9 |
| B3GNT6 | WBSCR17 | 9606.ENSP00000484640 | 9606.ENSP00000329654 | 0 | 0 | 0 | 0 | 0 | 0 | 0.65 | 0 | 0.65 |
| B3GNT6 | GALNT15 | 9606.ENSP00000484640 | 9606.ENSP00000344260 | 0 | 0 | 0 | 0 | 0 | 0 | 0.65 | 0 | 0.65 |
| B3GNT6 | GALNT2 | 9606.ENSP00000484640 | 9606.ENSP00000355632 | 0 | 0 | 0 | 0 | 0 | 0 | 0.65 | 0 | 0.65 |
| B3GNT6 | GALNT12 | 9606.ENSP00000484640 | 9606.ENSP00000364150 | 0 | 0 | 0 | 0 | 0 | 0 | 0.65 | 0 | 0.65 |
| B3GNT6 | GALNT3 | 9606.ENSP00000484640 | 9606.ENSP00000376465 | 0 | 0 | 0 | 0 | 0 | 0 | 0.65 | 0 | 0.65 |
| B3GNT6 | GCNT3 | 9606.ENSP00000484640 | 9606.ENSP00000379377 | 0 | 0 | 0 | 0 | 0 | 0 | 0.9 | 0 | 0.9 |
| B3GNT6 | GALNT9 | 9606.ENSP00000484640 | 9606.ENSP00000380488 | 0 | 0 | 0 | 0 | 0 | 0 | 0.65 | 0 | 0.65 |
| B3GNT6 | C1GALT1 | 9606.ENSP00000484640 | 9606.ENSP00000389176 | 0 | 0 | 0 | 0 | 0 | 0 | 0.9 | 0 | 0.9 |
| B3GNT6 | MUC6 | 9606.ENSP00000484640 | 9606.ENSP00000406861 | 0 | 0 | 0 | 0 | 0 | 0 | 0.9 | 0 | 0.9 |
| B3GNT6 | MUC7 | 9606.ENSP00000484640 | 9606.ENSP00000407422 | 0 | 0 | 0 | 0 | 0 | 0 | 0.9 | 0 | 0.9 |
| B3GNT6 | GCNT1 | 9606.ENSP00000484640 | 9606.ENSP00000415454 | 0 | 0 | 0 | 0 | 0 | 0 | 0.9 | 0 | 0.9 |
| B3GNT6 | GALNT11 | 9606.ENSP00000484640 | 9606.ENSP00000416787 | 0 | 0 | 0 | 0 | 0 | 0 | 0.65 | 0 | 0.65 |
| B3GNT6 | MUC4 | 9606.ENSP00000484640 | 9606.ENSP00000417498 | 0 | 0 | 0 | 0 | 0 | 0 | 0.9 | 0 | 0.9 |
| B3GNT6 | GALNT4 | 9606.ENSP00000484640 | 9606.ENSP00000436604 | 0 | 0 | 0 | 0 | 0 | 0 | 0.65 | 0 | 0.65 |
| B3GNT6 | MUC5B | 9606.ENSP00000484640 | 9606.ENSP00000436812 | 0 | 0 | 0 | 0 | 0 | 0 | 0.9 | 0 | 0.9 |
| B3GNT6 | GALNT6 | 9606.ENSP00000484640 | 9606.ENSP00000444171 | 0 | 0 | 0 | 0 | 0 | 0 | 0.65 | 0 | 0.65 |
| B3GNT6 | MUC13 | 9606.ENSP00000484640 | 9606.ENSP00000485028 | 0 | 0 | 0 | 0 | 0 | 0 | 0.9 | 0 | 0.9 |
| B3GNT6 | MUC5AC | 9606.ENSP00000484640 | 9606.ENSP00000485659 | 0 | 0 | 0 | 0 | 0 | 0 | 0.9 | 0 | 0.9 |
| B3GNT6 | MUC1 | 9606.ENSP00000484640 | 9606.ENSP00000484824 | 0 | 0 | 0 | 0 | 0 | 0 | 0.9 | 0 | 0.9 |
| B4GALT1 | ST6GAL1 | 9606.ENSP00000369055 | 9606.ENSP00000169298 | 0 | 0 | 0 | 0 | 0 | 0 | 0.9 | 0 | 0.9 |
| B4GALT1 | CEACAM6 | 9606.ENSP00000369055 | 9606.ENSP00000199764 | 0 | 0 | 0 | 0 | 0 | 0 | 0.9 | 0 | 0.9 |
| B4GALT1 | CMTM6 | 9606.ENSP00000369055 | 9606.ENSP00000205636 | 0 | 0 | 0 | 0 | 0 | 0 | 0.9 | 0 | 0.9 |
| B4GALT1 | SNAP29 | 9606.ENSP00000369055 | 9606.ENSP00000215730 | 0 | 0 | 0 | 0 | 0 | 0 | 0.9 | 0 | 0.9 |
| B4GALT1 | RAB3D | 9606.ENSP00000369055 | 9606.ENSP00000222120 | 0 | 0 | 0 | 0 | 0 | 0 | 0.9 | 0 | 0.9 |
| B4GALT1 | MANBA | 9606.ENSP00000369055 | 9606.ENSP00000226578 | 0 | 0 | 0 | 0 | 0 | 0 | 0.9 | 0 | 0.9 |
| B4GALT1 | TMEM30A | 9606.ENSP00000369055 | 9606.ENSP00000230461 | 0 | 0 | 0 | 0 | 0 | 0 | 0.9 | 0 | 0.9 |
| B4GALT1 | CEACAM8 | 9606.ENSP00000369055 | 9606.ENSP00000244336 | 0 | 0 | 0 | 0 | 0 | 0 | 0.9 | 0 | 0.9 |
| B4GALT1 | CD68 | 9606.ENSP00000369055 | 9606.ENSP00000250092 | 0 | 0 | 0 | 0 | 0 | 0 | 0.9 | 0 | 0.9 |
| B4GALT1 | RAP1B | 9606.ENSP00000369055 | 9606.ENSP00000250559 | 0 | 0 | 0 | 0 | 0 | 0 | 0.9 | 0 | 0.9 |
| B4GALT1 | NOTCH2 | 9606.ENSP00000369055 | 9606.ENSP00000256646 | 0 | 0 | 0 | 0 | 0 | 0 | 0.9 | 0 | 0.9 |
| B4GALT1 | ST3GAL3 | 9606.ENSP00000369055 | 9606.ENSP00000262915 | 0 | 0 | 0 | 0 | 0 | 0 | 0.9 | 0 | 0.9 |
| B4GALT1 | NOTCH3 | 9606.ENSP00000369055 | 9606.ENSP00000263388 | 0 | 0 | 0 | 0 | 0 | 0 | 0.9 | 0 | 0.9 |
| B4GALT1 | VAMP8 | 9606.ENSP00000369055 | 9606.ENSP00000263864 | 0 | 0 | 0 | 0 | 0 | 0 | 0.9 | 0 | 0.9 |
| B4GALT1 | KERA | 9606.ENSP00000369055 | 9606.ENSP00000266719 | 0 | 0 | 0 | 0 | 0 | 0 | 0.9 | 0 | 0.9 |
| B4GALT1 | ST8SIA2 | 9606.ENSP00000369055 | 9606.ENSP00000268164 | 0 | 0 | 0 | 0 | 0 | 0 | 0.9 | 0 | 0.9 |
| B4GALT1 | NOTCH1 | 9606.ENSP00000369055 | 9606.ENSP00000277541 | 0 | 0 | 0 | 0 | 0 | 0 | 0.9 | 0 | 0.9 |
| B4GALT1 | LALBA | 9606.ENSP00000369055 | 9606.ENSP00000301046 | 0 | 0 | 0 | 0 | 0 | 0.87 | 0.9 | 0 | 0.986 |
| B4GALT1 | FUT9 | 9606.ENSP00000369055 | 9606.ENSP00000302599 | 0 | 0 | 0 | 0 | 0 | 0 | 0.9 | 0 | 0.9 |
| B4GALT1 | FUT3 | 9606.ENSP00000369055 | 9606.ENSP00000305603 | 0 | 0 | 0 | 0 | 0 | 0 | 0.9 | 0 | 0.9 |
| B4GALT1 | GAA | 9606.ENSP00000369055 | 9606.ENSP00000305692 | 0 | 0 | 0 | 0 | 0 | 0.141 | 0.9 | 0 | 0.91 |
| B4GALT1 | B4GALT2 | 9606.ENSP00000369055 | 9606.ENSP00000310696 | 0 | 0 | 0 | 0.942 | 0 | 0 | 0.8 | 0 | 0.8 |
| B4GALT1 | FUT1 | 9606.ENSP00000369055 | 9606.ENSP00000312021 | 0 | 0 | 0 | 0 | 0 | 0 | 0.9 | 0 | 0.9 |
| B4GALT1 | FUT6 | 9606.ENSP00000369055 | 9606.ENSP00000313398 | 0 | 0 | 0 | 0 | 0 | 0 | 0.9 | 0 | 0.9 |
| B4GALT1 | FUT4 | 9606.ENSP00000369055 | 9606.ENSP00000351602 | 0 | 0 | 0 | 0 | 0 | 0 | 0.9 | 0 | 0.9 |
| B4GALT1 | FUT8 | 9606.ENSP00000369055 | 9606.ENSP00000353910 | 0 | 0 | 0 | 0 | 0 | 0 | 0.9 | 0 | 0.9 |
| B4GALT1 | POMGNT1 | 9606.ENSP00000369055 | 9606.ENSP00000361060 | 0 | 0 | 0 | 0 | 0 | 0 | 0.9 | 0 | 0.9 |
| B4GALT1 | NOTCH4 | 9606.ENSP00000369055 | 9606.ENSP00000364163 | 0 | 0 | 0 | 0 | 0 | 0 | 0.9 | 0 | 0.9 |
| B4GALT1 | GCNT2 | 9606.ENSP00000369055 | 9606.ENSP00000368917 | 0 | 0 | 0 | 0 | 0 | 0 | 0.9 | 0 | 0.9 |
| B4GALT1 | B4GALT3 | 9606.ENSP00000369055 | 9606.ENSP00000480428 | 0 | 0 | 0 | 0.916 | 0 | 0 | 0.8 | 0 | 0.8 |
| B4GALT1 | B4GALT4 | 9606.ENSP00000369055 | 9606.ENSP00000420161 | 0 | 0 | 0 | 0.911 | 0 | 0 | 0.8 | 0 | 0.8 |
| B4GALT1 | RAB5C | 9606.ENSP00000369055 | 9606.ENSP00000447053 | 0 | 0 | 0 | 0 | 0 | 0 | 0.9 | 0 | 0.9 |
| B4GALT1 | GCNT3 | 9606.ENSP00000369055 | 9606.ENSP00000379377 | 0 | 0 | 0 | 0 | 0 | 0 | 0.9 | 0 | 0.9 |
| B4GALT1 | FUT2 | 9606.ENSP00000369055 | 9606.ENSP00000387498 | 0 | 0 | 0 | 0 | 0 | 0 | 0.9 | 0 | 0.9 |
| B4GALT1 | MGAT5B | 9606.ENSP00000369055 | 9606.ENSP00000391227 | 0 | 0 | 0 | 0 | 0 | 0 | 0.9 | 0 | 0.9 |
| B4GALT1 | CDK11A | 9606.ENSP00000369055 | 9606.ENSP00000384442 | 0 | 0 | 0 | 0 | 0 | 0.379 | 0.9 | 0 | 0.935 |
| B4GALT1 | FUT5 | 9606.ENSP00000369055 | 9606.ENSP00000466880 | 0 | 0 | 0 | 0 | 0 | 0 | 0.9 | 0 | 0.9 |
| B4GALT1 | ST3GAL6 | 9606.ENSP00000369055 | 9606.ENSP00000480884 | 0 | 0 | 0 | 0 | 0 | 0 | 0.9 | 0 | 0.9 |
| B4GALT1 | ST6GAL2 | 9606.ENSP00000369055 | 9606.ENSP00000386942 | 0 | 0 | 0 | 0 | 0 | 0 | 0.9 | 0 | 0.9 |
| B4GALT2 | ST6GAL1 | 9606.ENSP00000310696 | 9606.ENSP00000169298 | 0 | 0 | 0 | 0 | 0 | 0 | 0.9 | 0 | 0.9 |
| B4GALT2 | ST3GAL3 | 9606.ENSP00000310696 | 9606.ENSP00000262915 | 0 | 0 | 0 | 0 | 0 | 0 | 0.9 | 0 | 0.9 |
| B4GALT2 | KERA | 9606.ENSP00000310696 | 9606.ENSP00000266719 | 0 | 0 | 0 | 0 | 0 | 0 | 0.9 | 0 | 0.9 |
| B4GALT2 | ST8SIA2 | 9606.ENSP00000310696 | 9606.ENSP00000268164 | 0 | 0 | 0 | 0 | 0 | 0 | 0.9 | 0 | 0.9 |
| B4GALT2 | LALBA | 9606.ENSP00000310696 | 9606.ENSP00000301046 | 0 | 0 | 0 | 0 | 0 | 0 | 0.8 | 0 | 0.8 |
| B4GALT2 | FUT9 | 9606.ENSP00000310696 | 9606.ENSP00000302599 | 0 | 0 | 0 | 0 | 0 | 0 | 0.9 | 0 | 0.9 |
| B4GALT2 | FUT3 | 9606.ENSP00000310696 | 9606.ENSP00000305603 | 0 | 0 | 0 | 0 | 0 | 0 | 0.9 | 0 | 0.9 |
| B4GALT2 | B4GALT3 | 9606.ENSP00000310696 | 9606.ENSP00000480428 | 0 | 0 | 0 | 0.906 | 0 | 0 | 0.8 | 0 | 0.8 |
| B4GALT2 | CDK11A | 9606.ENSP00000310696 | 9606.ENSP00000384442 | 0 | 0 | 0 | 0 | 0 | 0 | 0.9 | 0 | 0.9 |
| B4GALT2 | GCNT3 | 9606.ENSP00000310696 | 9606.ENSP00000379377 | 0 | 0 | 0 | 0 | 0 | 0 | 0.9 | 0 | 0.9 |
| B4GALT2 | FUT4 | 9606.ENSP00000310696 | 9606.ENSP00000351602 | 0 | 0 | 0 | 0 | 0 | 0 | 0.9 | 0 | 0.9 |
| B4GALT2 | POMGNT1 | 9606.ENSP00000310696 | 9606.ENSP00000361060 | 0 | 0 | 0 | 0 | 0 | 0 | 0.9 | 0 | 0.9 |
| B4GALT2 | FUT2 | 9606.ENSP00000310696 | 9606.ENSP00000387498 | 0 | 0 | 0 | 0 | 0 | 0 | 0.9 | 0 | 0.9 |
| B4GALT2 | ST3GAL6 | 9606.ENSP00000310696 | 9606.ENSP00000480884 | 0 | 0 | 0 | 0 | 0 | 0 | 0.9 | 0 | 0.9 |
| B4GALT2 | FUT5 | 9606.ENSP00000310696 | 9606.ENSP00000466880 | 0 | 0 | 0 | 0 | 0 | 0 | 0.9 | 0 | 0.9 |
| B4GALT2 | FUT1 | 9606.ENSP00000310696 | 9606.ENSP00000312021 | 0 | 0 | 0 | 0 | 0 | 0 | 0.9 | 0 | 0.9 |
| B4GALT2 | GCNT2 | 9606.ENSP00000310696 | 9606.ENSP00000368917 | 0 | 0 | 0 | 0 | 0 | 0 | 0.9 | 0 | 0.9 |
| B4GALT2 | FUT6 | 9606.ENSP00000310696 | 9606.ENSP00000313398 | 0 | 0 | 0 | 0 | 0 | 0 | 0.9 | 0 | 0.9 |
| B4GALT2 | MGAT5B | 9606.ENSP00000310696 | 9606.ENSP00000391227 | 0 | 0 | 0 | 0 | 0 | 0 | 0.9 | 0 | 0.9 |
| B4GALT2 | FUT8 | 9606.ENSP00000310696 | 9606.ENSP00000353910 | 0 | 0 | 0 | 0 | 0 | 0 | 0.9 | 0 | 0.9 |
| B4GALT2 | ST6GAL2 | 9606.ENSP00000310696 | 9606.ENSP00000386942 | 0 | 0 | 0 | 0 | 0 | 0 | 0.9 | 0 | 0.9 |
| FUT1 | FUT9 | 9606.ENSP00000312021 | 9606.ENSP00000302599 | 0 | 0 | 0 | 0 | 0 | 0 | 0.9 | 0 | 0.9 |
| FUT1 | FUT3 | 9606.ENSP00000312021 | 9606.ENSP00000305603 | 0 | 0 | 0 | 0 | 0 | 0 | 0.9 | 0 | 0.9 |
| FUT1 | FUT2 | 9606.ENSP00000312021 | 9606.ENSP00000387498 | 0 | 0 | 0 | 0.96 | 0 | 0 | 0.8 | 0 | 0.8 |
| FUT1 | GCNT2 | 9606.ENSP00000312021 | 9606.ENSP00000368917 | 0 | 0 | 0 | 0 | 0 | 0 | 0.9 | 0 | 0.9 |
| FUT1 | ST3GAL4 | 9606.ENSP00000312021 | 9606.ENSP00000436047 | 0 | 0 | 0 | 0 | 0 | 0 | 0.9 | 0 | 0.9 |
| FUT1 | ST3GAL6 | 9606.ENSP00000312021 | 9606.ENSP00000480884 | 0 | 0 | 0 | 0 | 0 | 0 | 0.9 | 0 | 0.9 |
| FUT1 | ST3GAL2 | 9606.ENSP00000312021 | 9606.ENSP00000377257 | 0 | 0 | 0 | 0 | 0 | 0 | 0.9 | 0 | 0.9 |
| FUT1 | ST3GAL1 | 9606.ENSP00000312021 | 9606.ENSP00000428540 | 0 | 0 | 0 | 0 | 0 | 0 | 0.9 | 0 | 0.9 |
| FUT1 | FUT4 | 9606.ENSP00000312021 | 9606.ENSP00000351602 | 0 | 0 | 0 | 0 | 0 | 0 | 0.9 | 0 | 0.9 |
| FUT1 | FUT6 | 9606.ENSP00000312021 | 9606.ENSP00000313398 | 0 | 0 | 0 | 0 | 0 | 0 | 0.9 | 0 | 0.9 |
| FUT1 | FUT5 | 9606.ENSP00000312021 | 9606.ENSP00000466880 | 0 | 0 | 0 | 0 | 0 | 0 | 0.9 | 0 | 0.9 |
| FUT3 | FUT9 | 9606.ENSP00000305603 | 9606.ENSP00000302599 | 0 | 0 | 0 | 0.877 | 0 | 0 | 0.9 | 0 | 0.9 |
| FUT3 | FUT6 | 9606.ENSP00000305603 | 9606.ENSP00000313398 | 0 | 0 | 0 | 0.98 | 0 | 0 | 0.8 | 0 | 0.8 |
| FUT3 | FUT5 | 9606.ENSP00000305603 | 9606.ENSP00000466880 | 0 | 0 | 0 | 0.983 | 0 | 0 | 0.8 | 0 | 0.8 |
| FUT3 | FUT4 | 9606.ENSP00000305603 | 9606.ENSP00000351602 | 0 | 0 | 0 | 0.877 | 0 | 0 | 0.9 | 0 | 0.9 |
| FUT3 | GCNT2 | 9606.ENSP00000305603 | 9606.ENSP00000368917 | 0 | 0 | 0 | 0 | 0 | 0 | 0.9 | 0 | 0.9 |
| FUT3 | ST8SIA1 | 9606.ENSP00000305603 | 9606.ENSP00000379353 | 0 | 0 | 0 | 0 | 0 | 0 | 0.9 | 0 | 0.9 |
| FUT3 | ST3GAL6 | 9606.ENSP00000305603 | 9606.ENSP00000480884 | 0 | 0 | 0 | 0 | 0 | 0 | 0.9 | 0 | 0.9 |
| FUT3 | ST3GAL4 | 9606.ENSP00000305603 | 9606.ENSP00000436047 | 0 | 0 | 0 | 0 | 0 | 0 | 0.9 | 0 | 0.9 |
| FUT9 | GCNT2 | 9606.ENSP00000302599 | 9606.ENSP00000368917 | 0 | 0 | 0 | 0 | 0 | 0 | 0.9 | 0 | 0.9 |
| FUT9 | ST3GAL6 | 9606.ENSP00000302599 | 9606.ENSP00000480884 | 0 | 0 | 0 | 0 | 0 | 0 | 0.9 | 0 | 0.9 |
| GALNT3 | ST6GALNAC1 | 9606.ENSP00000376465 | 9606.ENSP00000156626 | 0 | 0 | 0 | 0 | 0 | 0 | 0.65 | 0 | 0.65 |
| GALNT3 | GCNT1 | 9606.ENSP00000376465 | 9606.ENSP00000415454 | 0 | 0 | 0 | 0 | 0 | 0 | 0.65 | 0 | 0.65 |
| GALNT3 | MUC13 | 9606.ENSP00000376465 | 9606.ENSP00000485028 | 0 | 0 | 0 | 0 | 0 | 0 | 0.9 | 0 | 0.9 |
| GALNT3 | MUC5B | 9606.ENSP00000376465 | 9606.ENSP00000436812 | 0 | 0 | 0 | 0 | 0 | 0 | 0.9 | 0 | 0.9 |
| GALNT3 | MUC7 | 9606.ENSP00000376465 | 9606.ENSP00000407422 | 0 | 0 | 0 | 0 | 0 | 0 | 0.9 | 0 | 0.9 |
| GALNT3 | MUC6 | 9606.ENSP00000376465 | 9606.ENSP00000406861 | 0 | 0 | 0 | 0 | 0 | 0 | 0.9 | 0 | 0.9 |
| GALNT3 | MUC4 | 9606.ENSP00000376465 | 9606.ENSP00000417498 | 0 | 0 | 0 | 0 | 0 | 0 | 0.9 | 0 | 0.9 |
| GALNT3 | MUC5AC | 9606.ENSP00000376465 | 9606.ENSP00000485659 | 0 | 0 | 0 | 0 | 0 | 0 | 0.9 | 0 | 0.9 |
| GALNT3 | MUC1 | 9606.ENSP00000376465 | 9606.ENSP00000484824 | 0 | 0 | 0 | 0 | 0 | 0 | 0.9 | 0 | 0.9 |
| GCNT1 | ST6GALNAC1 | 9606.ENSP00000415454 | 9606.ENSP00000156626 | 0 | 0 | 0 | 0 | 0 | 0 | 0.9 | 0 | 0.9 |
| GCNT1 | GCNT4 | 9606.ENSP00000415454 | 9606.ENSP00000317027 | 0 | 0 | 0 | 0.9 | 0 | 0 | 0.8 | 0 | 0.8 |
| GCNT1 | WBSCR17 | 9606.ENSP00000415454 | 9606.ENSP00000329654 | 0 | 0 | 0 | 0 | 0 | 0 | 0.65 | 0 | 0.65 |
| GCNT1 | ST3GAL2 | 9606.ENSP00000415454 | 9606.ENSP00000377257 | 0 | 0 | 0 | 0 | 0 | 0 | 0.9 | 0 | 0.9 |
| GCNT1 | GCNT3 | 9606.ENSP00000415454 | 9606.ENSP00000379377 | 0 | 0 | 0 | 0.944 | 0 | 0 | 0.8 | 0 | 0.8 |
| GCNT1 | MUC6 | 9606.ENSP00000415454 | 9606.ENSP00000406861 | 0 | 0 | 0 | 0 | 0 | 0 | 0.9 | 0 | 0.9 |
| GCNT1 | MUC7 | 9606.ENSP00000415454 | 9606.ENSP00000407422 | 0 | 0 | 0 | 0 | 0 | 0 | 0.9 | 0 | 0.9 |
| GCNT1 | MUC5B | 9606.ENSP00000415454 | 9606.ENSP00000436812 | 0 | 0 | 0 | 0 | 0 | 0 | 0.9 | 0 | 0.9 |
| GCNT1 | MUC4 | 9606.ENSP00000415454 | 9606.ENSP00000417498 | 0 | 0 | 0 | 0 | 0 | 0 | 0.9 | 0 | 0.9 |
| GCNT1 | MUC5AC | 9606.ENSP00000415454 | 9606.ENSP00000485659 | 0 | 0 | 0 | 0 | 0 | 0 | 0.9 | 0 | 0.9 |
| GCNT1 | MUC13 | 9606.ENSP00000415454 | 9606.ENSP00000485028 | 0 | 0 | 0 | 0 | 0 | 0 | 0.9 | 0 | 0.9 |
| GCNT1 | MUC1 | 9606.ENSP00000415454 | 9606.ENSP00000484824 | 0 | 0 | 0 | 0 | 0 | 0 | 0.9 | 0 | 0.9 |
| GCNT1 | ST3GAL1 | 9606.ENSP00000415454 | 9606.ENSP00000428540 | 0 | 0 | 0 | 0 | 0 | 0 | 0.9 | 0 | 0.9 |
| MGAT4B | MGAT5B | 9606.ENSP00000338487 | 9606.ENSP00000391227 | 0 | 0 | 0 | 0 | 0 | 0 | 0.9 | 0 | 0.9 |
| MGAT4B | MGAT5 | 9606.ENSP00000338487 | 9606.ENSP00000386377 | 0 | 0 | 0 | 0 | 0 | 0 | 0.9 | 0 | 0.9 |
| POMGNT1 | POMT2 | 9606.ENSP00000361060 | 9606.ENSP00000261534 | 0 | 0 | 0 | 0 | 0 | 0 | 0.9 | 0 | 0.9 |
| POMGNT1 | POMT1 | 9606.ENSP00000361060 | 9606.ENSP00000361302 | 0 | 0 | 0 | 0 | 0 | 0 | 0.9 | 0 | 0.9 |
| A4GALT | B4GALT1 | 9606.ENSP00000384794 | 9606.ENSP00000369055 | 0 | 0 | 0 | 0 | 0 | 0 | 0.9 | 0 | 0.9 |
| B3GALNT1 | B4GALT1 | 9606.ENSP00000376532 | 9606.ENSP00000369055 | 0 | 0 | 0 | 0 | 0 | 0 | 0.9 | 0 | 0.9 |
| B3GALT1 | B4GALT1 | 9606.ENSP00000376456 | 9606.ENSP00000369055 | 0 | 0 | 0 | 0 | 0 | 0 | 0.9 | 0 | 0.9 |
| B3GALT2 | B4GALT1 | 9606.ENSP00000356404 | 9606.ENSP00000369055 | 0 | 0 | 0 | 0 | 0 | 0 | 0.9 | 0 | 0.9 |
| B3GALT5 | B4GALT1 | 9606.ENSP00000381699 | 9606.ENSP00000369055 | 0 | 0 | 0 | 0 | 0 | 0 | 0.9 | 0 | 0.9 |
| B3GAT1 | B4GALT1 | 9606.ENSP00000433847 | 9606.ENSP00000369055 | 0 | 0 | 0 | 0 | 0 | 0 | 0.9 | 0 | 0.9 |
| B3GAT2 | B4GALT1 | 9606.ENSP00000230053 | 9606.ENSP00000369055 | 0 | 0 | 0 | 0 | 0 | 0 | 0.9 | 0 | 0.9 |
| B3GNT1 | B4GALT1 | 9606.ENSP00000309096 | 9606.ENSP00000369055 | 0 | 0 | 0 | 0 | 0 | 0 | 0.9 | 0 | 0.9 |
| B3GNT3 | B4GALT1 | 9606.ENSP00000321874 | 9606.ENSP00000369055 | 0 | 0 | 0 | 0 | 0 | 0 | 0.9 | 0 | 0.9 |
| B3GNT4 | B4GALT1 | 9606.ENSP00000319636 | 9606.ENSP00000369055 | 0 | 0 | 0 | 0 | 0 | 0 | 0.9 | 0 | 0.9 |
| B3GNT5 | B4GALT1 | 9606.ENSP00000316173 | 9606.ENSP00000369055 | 0 | 0 | 0 | 0 | 0 | 0 | 0.9 | 0 | 0.9 |
| A4GALT | B4GALT2 | 9606.ENSP00000384794 | 9606.ENSP00000310696 | 0 | 0 | 0 | 0 | 0 | 0 | 0.9 | 0 | 0.9 |
| B3GALNT1 | B4GALT2 | 9606.ENSP00000376532 | 9606.ENSP00000310696 | 0 | 0 | 0 | 0 | 0 | 0 | 0.9 | 0 | 0.9 |
| B3GALT1 | B4GALT2 | 9606.ENSP00000376456 | 9606.ENSP00000310696 | 0 | 0 | 0 | 0 | 0 | 0 | 0.9 | 0 | 0.9 |
| B3GALT2 | B4GALT2 | 9606.ENSP00000356404 | 9606.ENSP00000310696 | 0 | 0 | 0 | 0 | 0 | 0 | 0.9 | 0 | 0.9 |
| B3GALT5 | B4GALT2 | 9606.ENSP00000381699 | 9606.ENSP00000310696 | 0 | 0 | 0 | 0 | 0 | 0 | 0.9 | 0 | 0.9 |
| B3GAT1 | B4GALT2 | 9606.ENSP00000433847 | 9606.ENSP00000310696 | 0 | 0 | 0 | 0 | 0 | 0 | 0.9 | 0 | 0.9 |
| B3GAT2 | B4GALT2 | 9606.ENSP00000230053 | 9606.ENSP00000310696 | 0 | 0 | 0 | 0 | 0 | 0 | 0.9 | 0 | 0.9 |
| B3GNT1 | B4GALT2 | 9606.ENSP00000309096 | 9606.ENSP00000310696 | 0 | 0 | 0 | 0 | 0 | 0 | 0.9 | 0 | 0.9 |
| B3GNT3 | B4GALT2 | 9606.ENSP00000321874 | 9606.ENSP00000310696 | 0 | 0 | 0 | 0 | 0 | 0 | 0.9 | 0 | 0.9 |
| B3GNT4 | B4GALT2 | 9606.ENSP00000319636 | 9606.ENSP00000310696 | 0 | 0 | 0 | 0 | 0 | 0 | 0.9 | 0 | 0.9 |
| B3GNT5 | B4GALT2 | 9606.ENSP00000316173 | 9606.ENSP00000310696 | 0 | 0 | 0 | 0 | 0 | 0 | 0.9 | 0 | 0.9 |
| B4GALT1 | B4GALT2 | 9606.ENSP00000369055 | 9606.ENSP00000310696 | 0 | 0 | 0 | 0.942 | 0 | 0 | 0.8 | 0 | 0.8 |
| ABO | FUT1 | 9606.ENSP00000483018 | 9606.ENSP00000312021 | 0 | 0 | 0 | 0 | 0 | 0 | 0.9 | 0 | 0.9 |
| B3GALT1 | FUT1 | 9606.ENSP00000376456 | 9606.ENSP00000312021 | 0 | 0 | 0 | 0 | 0 | 0 | 0.9 | 0 | 0.9 |
| B3GALT2 | FUT1 | 9606.ENSP00000356404 | 9606.ENSP00000312021 | 0 | 0 | 0 | 0 | 0 | 0 | 0.9 | 0 | 0.9 |
| B3GALT5 | FUT1 | 9606.ENSP00000381699 | 9606.ENSP00000312021 | 0 | 0 | 0 | 0 | 0 | 0 | 0.9 | 0 | 0.9 |
| B3GNT3 | FUT1 | 9606.ENSP00000321874 | 9606.ENSP00000312021 | 0 | 0 | 0 | 0 | 0 | 0 | 0.9 | 0 | 0.9 |
| B3GNT4 | FUT1 | 9606.ENSP00000319636 | 9606.ENSP00000312021 | 0 | 0 | 0 | 0 | 0 | 0 | 0.9 | 0 | 0.9 |
| B4GALT1 | FUT1 | 9606.ENSP00000369055 | 9606.ENSP00000312021 | 0 | 0 | 0 | 0 | 0 | 0 | 0.9 | 0 | 0.9 |
| B4GALT2 | FUT1 | 9606.ENSP00000310696 | 9606.ENSP00000312021 | 0 | 0 | 0 | 0 | 0 | 0 | 0.9 | 0 | 0.9 |
| B4GALT3 | FUT1 | 9606.ENSP00000480428 | 9606.ENSP00000312021 | 0 | 0 | 0 | 0 | 0 | 0 | 0.9 | 0 | 0.9 |
| B4GALT4 | FUT1 | 9606.ENSP00000420161 | 9606.ENSP00000312021 | 0 | 0 | 0 | 0 | 0 | 0 | 0.9 | 0 | 0.9 |
| ABO | FUT3 | 9606.ENSP00000483018 | 9606.ENSP00000305603 | 0 | 0 | 0 | 0 | 0 | 0 | 0.9 | 0 | 0.9 |
| B3GALT1 | FUT3 | 9606.ENSP00000376456 | 9606.ENSP00000305603 | 0 | 0 | 0 | 0 | 0 | 0 | 0.9 | 0 | 0.9 |
| B3GALT2 | FUT3 | 9606.ENSP00000356404 | 9606.ENSP00000305603 | 0 | 0 | 0 | 0 | 0 | 0 | 0.9 | 0 | 0.9 |
| B3GALT5 | FUT3 | 9606.ENSP00000381699 | 9606.ENSP00000305603 | 0 | 0 | 0 | 0 | 0 | 0 | 0.9 | 0 | 0.9 |
| B3GNT3 | FUT3 | 9606.ENSP00000321874 | 9606.ENSP00000305603 | 0 | 0 | 0 | 0 | 0 | 0 | 0.9 | 0 | 0.9 |
| B3GNT4 | FUT3 | 9606.ENSP00000319636 | 9606.ENSP00000305603 | 0 | 0 | 0 | 0 | 0 | 0 | 0.9 | 0 | 0.9 |
| B4GALT1 | FUT3 | 9606.ENSP00000369055 | 9606.ENSP00000305603 | 0 | 0 | 0 | 0 | 0 | 0 | 0.9 | 0 | 0.9 |
| B4GALT2 | FUT3 | 9606.ENSP00000310696 | 9606.ENSP00000305603 | 0 | 0 | 0 | 0 | 0 | 0 | 0.9 | 0 | 0.9 |
| B4GALT3 | FUT3 | 9606.ENSP00000480428 | 9606.ENSP00000305603 | 0 | 0 | 0 | 0 | 0 | 0 | 0.9 | 0 | 0.9 |
| B4GALT4 | FUT3 | 9606.ENSP00000420161 | 9606.ENSP00000305603 | 0 | 0 | 0 | 0 | 0 | 0 | 0.9 | 0 | 0.9 |
| FUT1 | FUT3 | 9606.ENSP00000312021 | 9606.ENSP00000305603 | 0 | 0 | 0 | 0 | 0 | 0 | 0.9 | 0 | 0.9 |
| FUT2 | FUT3 | 9606.ENSP00000387498 | 9606.ENSP00000305603 | 0 | 0 | 0 | 0 | 0 | 0 | 0.9 | 0 | 0.9 |
| ABO | FUT9 | 9606.ENSP00000483018 | 9606.ENSP00000302599 | 0 | 0 | 0 | 0 | 0 | 0 | 0.9 | 0 | 0.9 |
| B3GNT3 | FUT9 | 9606.ENSP00000321874 | 9606.ENSP00000302599 | 0 | 0 | 0 | 0 | 0 | 0 | 0.9 | 0 | 0.9 |
| B3GNT4 | FUT9 | 9606.ENSP00000319636 | 9606.ENSP00000302599 | 0 | 0 | 0 | 0 | 0 | 0 | 0.9 | 0 | 0.9 |
| B4GALT1 | FUT9 | 9606.ENSP00000369055 | 9606.ENSP00000302599 | 0 | 0 | 0 | 0 | 0 | 0 | 0.9 | 0 | 0.9 |
| B4GALT2 | FUT9 | 9606.ENSP00000310696 | 9606.ENSP00000302599 | 0 | 0 | 0 | 0 | 0 | 0 | 0.9 | 0 | 0.9 |
| B4GALT3 | FUT9 | 9606.ENSP00000480428 | 9606.ENSP00000302599 | 0 | 0 | 0 | 0 | 0 | 0 | 0.9 | 0 | 0.9 |
| B4GALT4 | FUT9 | 9606.ENSP00000420161 | 9606.ENSP00000302599 | 0 | 0 | 0 | 0 | 0 | 0 | 0.9 | 0 | 0.9 |
| FUT1 | FUT9 | 9606.ENSP00000312021 | 9606.ENSP00000302599 | 0 | 0 | 0 | 0 | 0 | 0 | 0.9 | 0 | 0.9 |
| FUT2 | FUT9 | 9606.ENSP00000387498 | 9606.ENSP00000302599 | 0 | 0 | 0 | 0 | 0 | 0 | 0.9 | 0 | 0.9 |
| FUT3 | FUT9 | 9606.ENSP00000305603 | 9606.ENSP00000302599 | 0 | 0 | 0 | 0.877 | 0 | 0 | 0.9 | 0 | 0.9 |
| FUT4 | FUT9 | 9606.ENSP00000351602 | 9606.ENSP00000302599 | 0 | 0 | 0 | 0.884 | 0 | 0 | 0.9 | 0 | 0.9 |
| FUT5 | FUT9 | 9606.ENSP00000466880 | 9606.ENSP00000302599 | 0 | 0 | 0 | 0.881 | 0 | 0 | 0.9 | 0 | 0.9 |
| FUT6 | FUT9 | 9606.ENSP00000313398 | 9606.ENSP00000302599 | 0 | 0 | 0 | 0.88 | 0 | 0 | 0.9 | 0 | 0.9 |
| B3GNT6 | GALNT3 | 9606.ENSP00000484640 | 9606.ENSP00000376465 | 0 | 0 | 0 | 0 | 0 | 0 | 0.65 | 0 | 0.65 |
| C1GALT1 | GALNT3 | 9606.ENSP00000389176 | 9606.ENSP00000376465 | 0 | 0 | 0 | 0 | 0 | 0 | 0.9 | 0 | 0.9 |
| C1GALT1C1 | GALNT3 | 9606.ENSP00000304364 | 9606.ENSP00000376465 | 0 | 0 | 0 | 0 | 0 | 0 | 0.65 | 0 | 0.65 |
| FGF23 | GALNT3 | 9606.ENSP00000237837 | 9606.ENSP00000376465 | 0 | 0 | 0 | 0 | 0 | 0 | 0.9 | 0 | 0.9 |
| B3GNT3 | GCNT1 | 9606.ENSP00000321874 | 9606.ENSP00000415454 | 0 | 0 | 0 | 0 | 0 | 0 | 0.9 | 0 | 0.9 |
| B3GNT6 | GCNT1 | 9606.ENSP00000484640 | 9606.ENSP00000415454 | 0 | 0 | 0 | 0 | 0 | 0 | 0.9 | 0 | 0.9 |
| B4GALT5 | GCNT1 | 9606.ENSP00000360776 | 9606.ENSP00000415454 | 0 | 0 | 0 | 0 | 0 | 0 | 0.9 | 0 | 0.9 |
| C1GALT1 | GCNT1 | 9606.ENSP00000389176 | 9606.ENSP00000415454 | 0 | 0 | 0 | 0 | 0 | 0 | 0.9 | 0 | 0.9 |
| C1GALT1C1 | GCNT1 | 9606.ENSP00000304364 | 9606.ENSP00000415454 | 0 | 0 | 0 | 0 | 0 | 0 | 0.9 | 0 | 0.9 |
| GALNT1 | GCNT1 | 9606.ENSP00000269195 | 9606.ENSP00000415454 | 0 | 0 | 0 | 0 | 0 | 0 | 0.65 | 0 | 0.65 |
| GALNT10 | GCNT1 | 9606.ENSP00000297107 | 9606.ENSP00000415454 | 0 | 0 | 0 | 0 | 0 | 0 | 0.65 | 0 | 0.65 |
| GALNT11 | GCNT1 | 9606.ENSP00000416787 | 9606.ENSP00000415454 | 0 | 0 | 0 | 0 | 0 | 0 | 0.65 | 0 | 0.65 |
| GALNT12 | GCNT1 | 9606.ENSP00000364150 | 9606.ENSP00000415454 | 0 | 0 | 0 | 0 | 0 | 0 | 0.65 | 0 | 0.65 |
| GALNT15 | GCNT1 | 9606.ENSP00000344260 | 9606.ENSP00000415454 | 0 | 0 | 0 | 0 | 0 | 0 | 0.65 | 0 | 0.65 |
| GALNT18 | GCNT1 | 9606.ENSP00000227756 | 9606.ENSP00000415454 | 0 | 0 | 0 | 0 | 0 | 0 | 0.65 | 0 | 0.65 |
| GALNT2 | GCNT1 | 9606.ENSP00000355632 | 9606.ENSP00000415454 | 0 | 0 | 0 | 0 | 0 | 0 | 0.65 | 0 | 0.65 |
| GALNT3 | GCNT1 | 9606.ENSP00000376465 | 9606.ENSP00000415454 | 0 | 0 | 0 | 0 | 0 | 0 | 0.65 | 0 | 0.65 |
| GALNT4 | GCNT1 | 9606.ENSP00000436604 | 9606.ENSP00000415454 | 0 | 0 | 0 | 0 | 0 | 0 | 0.65 | 0 | 0.65 |
| GALNT5 | GCNT1 | 9606.ENSP00000259056 | 9606.ENSP00000415454 | 0 | 0 | 0 | 0 | 0 | 0 | 0.65 | 0 | 0.65 |
| GALNT6 | GCNT1 | 9606.ENSP00000444171 | 9606.ENSP00000415454 | 0 | 0 | 0 | 0 | 0 | 0 | 0.65 | 0 | 0.65 |
| GALNT7 | GCNT1 | 9606.ENSP00000265000 | 9606.ENSP00000415454 | 0 | 0 | 0 | 0 | 0 | 0 | 0.65 | 0 | 0.65 |
| GALNT8 | GCNT1 | 9606.ENSP00000252318 | 9606.ENSP00000415454 | 0 | 0 | 0 | 0 | 0 | 0 | 0.65 | 0 | 0.65 |
| GALNT9 | GCNT1 | 9606.ENSP00000380488 | 9606.ENSP00000415454 | 0 | 0 | 0 | 0 | 0 | 0 | 0.65 | 0 | 0.65 |
| FUT8 | MGAT4B | 9606.ENSP00000353910 | 9606.ENSP00000338487 | 0 | 0 | 0 | 0 | 0 | 0 | 0.9 | 0 | 0.9 |
| MGAT2 | MGAT4B | 9606.ENSP00000307423 | 9606.ENSP00000338487 | 0 | 0 | 0 | 0 | 0 | 0 | 0.9 | 0 | 0.9 |
| MGAT3 | MGAT4B | 9606.ENSP00000345270 | 9606.ENSP00000338487 | 0 | 0 | 0 | 0 | 0 | 0 | 0.9 | 0 | 0.9 |
| MGAT4A | MGAT4B | 9606.ENSP00000264968 | 9606.ENSP00000338487 | 0 | 0 | 0 | 0.96 | 0 | 0 | 0.8 | 0 | 0.8 |
| B4GALT1 | POMGNT1 | 9606.ENSP00000369055 | 9606.ENSP00000361060 | 0 | 0 | 0 | 0 | 0 | 0 | 0.9 | 0 | 0.9 |
| B4GALT2 | POMGNT1 | 9606.ENSP00000310696 | 9606.ENSP00000361060 | 0 | 0 | 0 | 0 | 0 | 0 | 0.9 | 0 | 0.9 |
| B4GALT3 | POMGNT1 | 9606.ENSP00000480428 | 9606.ENSP00000361060 | 0 | 0 | 0 | 0 | 0 | 0.26 | 0.9 | 0 | 0.922 |
| DAG1 | POMGNT1 | 9606.ENSP00000442600 | 9606.ENSP00000361060 | 0 | 0 | 0 | 0 | 0 | 0 | 0.9 | 0 | 0.9 |
| MGAT5B | POMGNT1 | 9606.ENSP00000391227 | 9606.ENSP00000361060 | 0 | 0 | 0 | 0 | 0 | 0 | 0.9 | 0 | 0.9 |

eTable 2. Results of cytoHubba calculation.

| Gene | Betweenness | Bottle Neck | Closeness | Clustering Coefficient | Degree | DMNC | EcCentricity | EPC | MCC | MNC | Radiality | Stress |
| --- | --- | --- | --- | --- | --- | --- | --- | --- | --- | --- | --- | --- |
| GPC3 | 22694.62127 | 68 | 180.04286 | 0.33107 | 60 | 0.55596 | 0.14075 | 121.8 | 9.22E+13 | 60 | 11.094 | 481664 |
| B4GALT1 | 20100.79474 | 20 | 175.20992 | 0.19673 | 50 | 0.36092 | 0.10947 | 104.517 | 2.40E+09 | 38 | 11.00041 | 816204 |
| MUC6 | 2219.86055 | 8 | 148.59722 | 0.35253 | 45 | 0.53996 | 0.09853 | 102.715 | 7.67E+09 | 45 | 10.34028 | 200328 |
| MUC5AC | 2219.86055 | 1 | 148.59722 | 0.35253 | 45 | 0.53996 | 0.09853 | 104.485 | 7.67E+09 | 45 | 10.34028 | 200328 |
| MUC5B | 2219.86055 | 1 | 148.59722 | 0.35253 | 45 | 0.53996 | 0.09853 | 101.418 | 7.67E+09 | 45 | 10.34028 | 200328 |
| MUC13 | 2219.86055 | 1 | 148.59722 | 0.35253 | 45 | 0.53996 | 0.09853 | 102.127 | 7.67E+09 | 45 | 10.34028 | 200328 |
| MUC4 | 2219.86055 | 4 | 148.59722 | 0.35253 | 45 | 0.53996 | 0.09853 | 102.287 | 7.67E+09 | 45 | 10.34028 | 200328 |
| MUC1 | 2219.86055 | 4 | 148.59722 | 0.35253 | 45 | 0.53996 | 0.09853 | 103.608 | 7.67E+09 | 45 | 10.34028 | 200328 |
| MUC7 | 2219.86055 | 2 | 148.59722 | 0.35253 | 45 | 0.53996 | 0.09853 | 102.52 | 7.67E+09 | 45 | 10.34028 | 200328 |
| C3 | 9883.23789 | 27 | 168.73929 | 0.43522 | 43 | 0.6569 | 0.12316 | 114.383 | 9.22E+13 | 43 | 10.93636 | 104860 |
| HSPG2 | 5228.25109 | 2 | 159.50952 | 0.31127 | 42 | 0.46624 | 0.14075 | 110.563 | 5.57E+07 | 42 | 10.78857 | 307928 |
| AGRN | 4833.78472 | 1 | 157.43571 | 0.30778 | 42 | 0.4803 | 0.14075 | 109.58 | 5.57E+07 | 41 | 10.73192 | 288612 |
| APOB | 7203.36589 | 6 | 171.83929 | 0.50976 | 41 | 0.75761 | 0.12316 | 117.871 | 9.22E+13 | 41 | 11.0275 | 279820 |
| FGF23 | 12665.72384 | 56 | 174.43929 | 0.47821 | 40 | 0.73604 | 0.12316 | 116.133 | 9.22E+13 | 39 | 11.08169 | 308850 |
| LAMC1 | 2708.76497 | 2 | 168.20595 | 0.55601 | 39 | 0.81299 | 0.12316 | 116.448 | 9.22E+13 | 39 | 10.95114 | 101152 |
| LAMB1 | 2708.76497 | 1 | 168.20595 | 0.55601 | 39 | 0.81299 | 0.12316 | 117.446 | 9.22E+13 | 39 | 10.95114 | 101152 |
| BMP4 | 5385.07299 | 11 | 167.37262 | 0.56006 | 37 | 1.23044 | 0.12316 | 116.677 | 9.22E+13 | 28 | 10.94622 | 76192 |
| GPC1 | 3893.1723 | 1 | 157.00952 | 0.35135 | 37 | 0.50498 | 0.14075 | 105.507 | 5.56E+07 | 37 | 10.77133 | 232042 |
| APOA1 | 1007.01149 | 1 | 160.85595 | 0.66723 | 35 | 0.98918 | 0.12316 | 117.223 | 9.22E+13 | 34 | 10.83291 | 23432 |
| APOE | 3676.87102 | 3 | 146.47103 | 0.32086 | 34 | 0.4485 | 0.09853 | 83.851 | 7999538 | 34 | 10.42649 | 264510 |
| GPC5 | 1728.71154 | 1 | 149.71905 | 0.40642 | 34 | 0.56809 | 0.14075 | 101.756 | 5.56E+07 | 34 | 10.6334 | 165430 |
| GPC6 | 1728.71154 | 1 | 149.71905 | 0.40642 | 34 | 0.56809 | 0.14075 | 102.217 | 5.56E+07 | 34 | 10.6334 | 165430 |
| GPC4 | 1728.71154 | 1 | 149.71905 | 0.40642 | 34 | 0.56809 | 0.14075 | 102.554 | 5.56E+07 | 34 | 10.6334 | 165430 |
| GPC2 | 1728.71154 | 2 | 149.71905 | 0.40642 | 34 | 0.56809 | 0.14075 | 104.857 | 5.56E+07 | 34 | 10.6334 | 165430 |
| KAT2B | 357.01843 | 1 | 158.60595 | 0.70766 | 34 | 0.98918 | 0.12316 | 115.886 | 9.22E+13 | 34 | 10.80089 | 7518 |
| B4GALT3 | 5656.39949 | 32 | 152.92659 | 0.2852 | 34 | 0.41942 | 0.10947 | 93.729 | 1.92E+09 | 33 | 10.64571 | 569056 |
| FUCA2 | 1876.44836 | 5 | 138.92103 | 0.32955 | 33 | 0.45612 | 0.09853 | 75.342 | 4099068 | 33 | 10.19496 | 74532 |
| MGAT4A | 3426.3359 | 7 | 161.00595 | 0.68182 | 33 | 1.29416 | 0.12316 | 113.176 | 9.22E+13 | 27 | 10.88464 | 31830 |
| WDR5 | 4279.54781 | 4 | 163.07262 | 0.68939 | 33 | 1.00542 | 0.12316 | 113.019 | 9.22E+13 | 32 | 10.89942 | 38106 |
| B4GALT2 | 4094.36051 | 2 | 152.09325 | 0.26894 | 33 | 0.37223 | 0.10947 | 90.974 | 9.58E+08 | 33 | 10.63832 | 398468 |
| SERPIND1 | 760.08652 | 16 | 138.18056 | 0.55242 | 32 | 0.75683 | 0.09853 | 96.569 | 7.66E+09 | 32 | 10.22205 | 66998 |
| HSP90B1 | 588.06069 | 2 | 136.08056 | 0.55645 | 32 | 0.76235 | 0.09853 | 97.331 | 7.67E+09 | 32 | 10.14077 | 44394 |
| GCNT1 | 5304.33067 | 17 | 161.52262 | 0.72177 | 32 | 1.15918 | 0.12316 | 115.603 | 9.22E+13 | 29 | 10.86247 | 56666 |
| C1GALT1 | 2435.21449 | 3 | 164.03929 | 0.72177 | 32 | 1.16898 | 0.12316 | 113.444 | 9.22E+13 | 29 | 10.92158 | 37884 |
| DCN | 11385.22892 | 18 | 151.22262 | 0.2621 | 32 | 0.53751 | 0.12316 | 84.128 | 5.08E+07 | 23 | 10.6334 | 218550 |
| PROC | 5019.4413 | 4 | 157.69325 | 0.13763 | 31 | 0.23597 | 0.10947 | 81.718 | 228 | 27 | 10.75409 | 105584 |
| RAF1 | 806.52016 | 2 | 158.43929 | 0.77419 | 31 | 1.04951 | 0.12316 | 113.098 | 9.22E+13 | 31 | 10.81567 | 9010 |
| TIMP1 | 471.99571 | 1 | 133.74722 | 0.5977 | 30 | 0.80143 | 0.09853 | 97.743 | 7.66E+09 | 30 | 10.11614 | 35352 |
| C1GALT1C1 | 264.68471 | 1 | 156.52262 | 0.82299 | 30 | 1.10351 | 0.12316 | 112.433 | 9.22E+13 | 30 | 10.78857 | 4730 |
| IGFBP5 | 113.32447 | 1 | 156.02262 | 0.87438 | 29 | 1.15918 | 0.12316 | 110.072 | 9.22E+13 | 29 | 10.78611 | 2262 |
| LGALS1 | 6363.10698 | 3 | 158.94325 | 0.14286 | 28 | 0.29615 | 0.10947 | 92.81 | 376 | 16 | 10.83784 | 329648 |
| FSTL3 | 5760.59249 | 8 | 158.80437 | 0.35185 | 28 | 0.46098 | 0.09853 | 87.447 | 4049046 | 28 | 10.74916 | 405444 |
| NOTCH1 | 2501.11496 | 9 | 148.2877 | 0.30688 | 28 | 0.40206 | 0.09853 | 80.807 | 4002242 | 28 | 10.52994 | 80112 |
| HDAC1 | 402.87273 | 4 | 160.17262 | 0.93122 | 28 | 1.22004 | 0.12316 | 114.067 | 9.22E+13 | 28 | 10.89942 | 7448 |
| Pathways in cancer | 523.39935 | 2 | 155.27262 | 0.92857 | 28 | 1.29416 | 0.12316 | 111.842 | 9.22E+13 | 27 | 10.77872 | 5120 |
| VGF | 4862.53155 | 3 | 150.02659 | 0.20798 | 27 | 0.3561 | 0.10947 | 62.484 | 5863 | 21 | 10.59645 | 82558 |
| P4HB | 362.07351 | 1 | 130.41389 | 0.64103 | 27 | 0.82959 | 0.09853 | 93.955 | 1.44E+09 | 27 | 10.07426 | 25590 |
| WFS1 | 1809.81375 | 1 | 137.7877 | 0.34758 | 27 | 0.44982 | 0.09853 | 69.277 | 95112 | 27 | 10.24176 | 73640 |
| BPIFB2 | 7205.93585 | 12 | 155.82103 | 0.28205 | 27 | 0.36502 | 0.09853 | 70.423 | 4.79E+08 | 27 | 10.71714 | 110802 |
| SCG3 | 0 | 1 | 154.77262 | 1 | 27 | 1.29416 | 0.12316 | 113.702 | 9.22E+13 | 27 | 10.77626 | 0 |
| EVA1A | 0 | 1 | 154.77262 | 1 | 27 | 1.29416 | 0.12316 | 113.028 | 9.22E+13 | 27 | 10.77626 | 0 |
| SPP2 | 0 | 1 | 154.77262 | 1 | 27 | 1.29416 | 0.12316 | 111.098 | 9.22E+13 | 27 | 10.77626 | 0 |
| RCN1 | 0 | 1 | 154.77262 | 1 | 27 | 1.29416 | 0.12316 | 111.357 | 9.22E+13 | 27 | 10.77626 | 0 |
| TMEM132A | 0 | 1 | 154.77262 | 1 | 27 | 1.29416 | 0.12316 | 109.246 | 9.22E+13 | 27 | 10.77626 | 0 |
| ANO8 | 0 | 1 | 154.77262 | 1 | 27 | 1.29416 | 0.12316 | 109.472 | 9.22E+13 | 27 | 10.77626 | 0 |
| B3GNT6 | 0 | 1 | 154.77262 | 1 | 27 | 1.29416 | 0.12316 | 110.774 | 9.22E+13 | 27 | 10.77626 | 0 |
| HCFC1 | 0 | 1 | 154.77262 | 1 | 27 | 1.29416 | 0.12316 | 113.321 | 9.22E+13 | 27 | 10.77626 | 0 |
| RAP1B | 0 | 1 | 154.77262 | 1 | 27 | 1.29416 | 0.12316 | 113.28 | 9.22E+13 | 27 | 10.77626 | 0 |
| PPP1CA | 0 | 1 | 154.77262 | 1 | 27 | 1.29416 | 0.12316 | 110.814 | 9.22E+13 | 27 | 10.77626 | 0 |
| B3GNT3 | 1737.51966 | 8 | 141.55437 | 0.41667 | 25 | 0.52531 | 0.09853 | 73.839 | 4365530 | 25 | 10.39693 | 117018 |
| KAT2A | 4218.6113 | 23 | 157.8877 | 0.34333 | 25 | 0.46396 | 0.09853 | 83.139 | 3675937 | 24 | 10.76148 | 303332 |
| NOTCH3 | 3546.27934 | 1 | 156.05437 | 0.36333 | 25 | 0.45807 | 0.09853 | 81.878 | 3680976 | 25 | 10.72207 | 290728 |
| NOTCH2 | 3546.27934 | 1 | 156.05437 | 0.36333 | 25 | 0.45807 | 0.09853 | 81.082 | 3680976 | 25 | 10.72207 | 290728 |
| NOTCH4 | 1299.10706 | 1 | 139.9377 | 0.5 | 25 | 0.63037 | 0.09853 | 91.063 | 1.92E+09 | 25 | 10.31072 | 100714 |
| ST3GAL1 | 3381.97796 | 9 | 141.04325 | 0.25 | 24 | 0.31081 | 0.10947 | 52.743 | 5760 | 24 | 10.40432 | 47390 |
| ST3GAL2 | 1207.72406 | 5 | 124.3925 | 0.42029 | 24 | 0.52252 | 0.08957 | 86.791 | 6.23E+09 | 24 | 9.73928 | 253924 |
| UBA52 | 1207.72406 | 1 | 124.3925 | 0.42029 | 24 | 0.52252 | 0.08957 | 86.676 | 6.23E+09 | 24 | 9.73928 | 253924 |
| PPP1CB | 4959.04325 | 8 | 160.42659 | 0.25362 | 24 | 0.33897 | 0.10947 | 78.619 | 8677 | 23 | 10.86001 | 224120 |
| RPS27A | 4161.04325 | 1 | 159.92659 | 0.27668 | 23 | 0.33897 | 0.10947 | 76.733 | 8676 | 23 | 10.85754 | 214126 |
| GALNT1 | 1391.47167 | 8 | 145.12103 | 0.2987 | 22 | 0.36035 | 0.09853 | 73.929 | 368180 | 22 | 10.5127 | 39414 |
| GALNT2 | 1861.39628 | 5 | 143.24325 | 0.47186 | 22 | 0.56926 | 0.10947 | 68.798 | 3690732 | 22 | 10.47822 | 56070 |
| RBBP5 | 3027.53636 | 4 | 156.40992 | 0.37229 | 22 | 0.52814 | 0.10947 | 73.307 | 4.79E+08 | 20 | 10.82306 | 58626 |
| VAMP8 | 2487.55381 | 8 | 156.07659 | 0.44156 | 22 | 0.5327 | 0.10947 | 69.781 | 4.79E+08 | 22 | 10.82306 | 63696 |
| RAB5C | 1525.01243 | 1 | 129.81389 | 0.50216 | 22 | 0.60581 | 0.09853 | 83.63 | 4.83E+08 | 22 | 10.10628 | 130034 |
| HDAC2 | 1525.01243 | 1 | 129.81389 | 0.50216 | 22 | 0.60581 | 0.09853 | 83.584 | 4.83E+08 | 22 | 10.10628 | 130034 |
| FUT2 | 405.97645 | 1 | 125.81389 | 0.35714 | 21 | 0.42392 | 0.09853 | 85.567 | 2880 | 21 | 10.01761 | 27978 |
| FUT1 | 2671.61577 | 13 | 142.90437 | 0.52857 | 21 | 0.62741 | 0.09853 | 83.755 | 1.92E+09 | 21 | 10.47083 | 118356 |
| ST3GAL6 | 870.92201 | 1 | 137.77103 | 0.52857 | 21 | 0.62741 | 0.09853 | 86.606 | 9.58E+08 | 21 | 10.29841 | 78990 |
| B3GNT4 | 548.5339 | 1 | 132.6877 | 0.6 | 21 | 0.71219 | 0.09853 | 78.299 | 1.92E+09 | 21 | 10.19988 | 130546 |
| ST6GALNAC1 | 548.5339 | 1 | 132.6877 | 0.6 | 21 | 0.71219 | 0.09853 | 79.893 | 1.92E+09 | 21 | 10.19988 | 130546 |
| FUT3 | 1288.93528 | 16 | 137.45992 | 0.34737 | 20 | 0.40532 | 0.10947 | 50.396 | 5744 | 20 | 10.38708 | 27222 |
| B4GALT4 | 1887.77434 | 12 | 140.15992 | 0.51053 | 20 | 0.59569 | 0.10947 | 61.427 | 3681006 | 20 | 10.43635 | 39386 |
| KMT2A | 325.50452 | 4 | 125.4377 | 0.47368 | 20 | 0.5527 | 0.09853 | 59.574 | 444360 | 20 | 9.99544 | 23984 |
| ACTR10 | 1375.55553 | 4 | 146.69325 | 0.21053 | 20 | 0.24565 | 0.10947 | 59.483 | 170 | 20 | 10.63832 | 39384 |
| KAT8 | 3276.76739 | 4 | 143.30992 | 0.50526 | 20 | 0.58955 | 0.10947 | 65.334 | 5443322 | 20 | 10.57182 | 56238 |
| PPP1CC | 293.28446 | 1 | 131.52103 | 0.65789 | 20 | 0.76764 | 0.09853 | 80.105 | 1.92E+09 | 20 | 10.18757 | 49830 |
| BRAF | 131.40256 | 1 | 134.00437 | 0.58421 | 20 | 0.68167 | 0.09853 | 77.427 | 9.58E+08 | 20 | 10.26393 | 3236 |
| ST3GAL4 | 4745.15358 | 7 | 137.1377 | 0.15789 | 19 | 0.62053 | 0.09853 | 69.618 | 1498 | 9 | 10.33043 | 238600 |
| KERA | 3686.70683 | 6 | 136.82583 | 0.28655 | 19 | 0.8164 | 0.08957 | 84.99 | 41065 | 8 | 10.21959 | 390106 |
| GCNT3 | 3851.20389 | 7 | 149.72659 | 0.32026 | 18 | 0.77815 | 0.10947 | 76.499 | 45483 | 10 | 10.71468 | 201422 |
| DCTN2 | 2065.83496 | 1 | 146.30992 | 0.23529 | 18 | 0.26445 | 0.10947 | 66.094 | 88 | 18 | 10.57428 | 21238 |
| ASH2L | 2070.77686 | 4 | 144.27103 | 0.54248 | 18 | 0.67192 | 0.09853 | 56.712 | 4.79E+08 | 17 | 10.55458 | 35916 |
| SNAP29 | 1510.33789 | 1 | 135.60437 | 0.6732 | 18 | 0.75661 | 0.09853 | 84.91 | 6.27E+09 | 18 | 10.27624 | 189534 |
| YY1 | 2459.05196 | 7 | 136.60437 | 0.34641 | 18 | 0.47563 | 0.09853 | 50.568 | 40838 | 16 | 10.33289 | 33408 |
| OGT | 739.75349 | 1 | 134.55437 | 0.42484 | 18 | 0.47747 | 0.09853 | 58.112 | 6024 | 18 | 10.30334 | 46424 |
| Human papillomavirus infection | 1175.60352 | 1 | 140.15992 | 0.57516 | 18 | 0.64643 | 0.10947 | 63.167 | 3650286 | 18 | 10.4462 | 31372 |
| IGF1 | 1037.04972 | 2 | 135.27103 | 0.60131 | 18 | 0.67581 | 0.09853 | 57.245 | 5443328 | 18 | 10.36738 | 56692 |
| FUT4 | 2199.76406 | 4 | 142.35992 | 0.21324 | 17 | 0.3366 | 0.10947 | 65.388 | 187 | 12 | 10.56936 | 84174 |
| ACTR1A | 1217.19659 | 1 | 147.64325 | 0.22794 | 17 | 0.2782 | 0.10947 | 63.504 | 115 | 16 | 10.65557 | 43820 |
| ARAF | 932.41649 | 4 | 134.35437 | 0.64706 | 17 | 0.71239 | 0.09853 | 56.75 | 5443208 | 17 | 10.35506 | 53352 |
| PI3K-Akt signaling pathway | 433.92302 | 1 | 129.6877 | 0.74265 | 17 | 0.81763 | 0.09853 | 69.757 | 1.92E+09 | 17 | 10.17525 | 50512 |
| FUT5 | 2476.66789 | 1 | 147.94325 | 0.23333 | 16 | 0.35915 | 0.10947 | 61.072 | 269 | 10 | 10.67034 | 39944 |
| FUT6 | 2136.12784 | 8 | 146.10437 | 0.61667 | 16 | 0.7411 | 0.09853 | 57.828 | 4.79E+08 | 15 | 10.60877 | 45042 |
| DCTN4 | 203.32934 | 1 | 127.17103 | 0.74167 | 16 | 0.7987 | 0.09853 | 53.925 | 5443320 | 16 | 10.1654 | 22136 |
| DCTN1 | 203.32934 | 1 | 127.17103 | 0.74167 | 16 | 0.7987 | 0.09853 | 54.475 | 5443320 | 16 | 10.1654 | 22136 |
| DCTN5 | 203.32934 | 1 | 127.17103 | 0.74167 | 16 | 0.7987 | 0.09853 | 51.527 | 5443320 | 16 | 10.1654 | 22136 |
| DCTN6 | 324.79979 | 1 | 129.92103 | 0.725 | 16 | 0.78076 | 0.09853 | 52.61 | 5443206 | 16 | 10.25407 | 23746 |
| DCTN3 | 203.32934 | 1 | 127.17103 | 0.74167 | 16 | 0.7987 | 0.09853 | 50.032 | 5443320 | 16 | 10.1654 | 22136 |
| GAA | 224.49817 | 2 | 129.1877 | 0.825 | 16 | 0.88845 | 0.09853 | 72.352 | 1.92E+09 | 16 | 10.17279 | 38192 |
| Focal adhesion | 224.49817 | 1 | 129.1877 | 0.825 | 16 | 0.88845 | 0.09853 | 71.353 | 1.92E+09 | 16 | 10.17279 | 38192 |
| FUT9 | 5565.91897 | 1 | 147.75595 | 0.25714 | 15 | 0.2704 | 0.12316 | 66.756 | 166 | 15 | 10.66295 | 86520 |
| B3GALT5 | 757.06087 | 6 | 131.94325 | 0.27619 | 15 | 0.29043 | 0.10947 | 49.969 | 188 | 15 | 10.31072 | 9342 |
| RNF2 | 1299.7281 | 10 | 145.33929 | 0.19048 | 15 | 0.22522 | 0.12316 | 55.736 | 45 | 14 | 10.67773 | 35390 |
| B3GNT5 | 3203.91537 | 1 | 143.57103 | 0.35238 | 15 | 0.46652 | 0.09853 | 81.415 | 5184 | 8 | 10.51763 | 387016 |
| WDR82 | 822.17947 | 2 | 135.9377 | 0.45714 | 15 | 0.8164 | 0.09853 | 72.879 | 41760 | 8 | 10.33289 | 71550 |
| ST3GAL3 | 813.95721 | 2 | 131.3377 | 0.53333 | 15 | 0.56083 | 0.09853 | 64.129 | 82874 | 15 | 10.24422 | 118914 |
| Complement and coagulation cascades | 169.4221 | 1 | 125.00437 | 0.53333 | 15 | 0.56083 | 0.09853 | 55.098 | 41604 | 15 | 10.03732 | 9348 |
| Proteoglycans in cancer | 469.11329 | 1 | 122.29008 | 0.45714 | 15 | 0.48071 | 0.09853 | 42.881 | 10116 | 15 | 9.86736 | 5094 |
| ITGAM | 1682.00176 | 5 | 143.84325 | 0.2381 | 15 | 0.31933 | 0.10947 | 63.409 | 744 | 13 | 10.62601 | 37710 |
| FGFR1 | 98.04619 | 1 | 128.1877 | 0.90476 | 15 | 0.95141 | 0.09853 | 66.56 | 1.92E+09 | 15 | 10.16294 | 17414 |
| GCNT4 | 1477.96077 | 7 | 134.52103 | 0.46154 | 14 | 0.85919 | 0.09853 | 56.002 | 362892 | 9 | 10.35999 | 58128 |
| MAML3 | 520.45497 | 1 | 129.22659 | 0.50549 | 14 | 0.51801 | 0.10947 | 42.006 | 5568 | 14 | 10.25407 | 13762 |
| MAML2 | 41.67992 | 2 | 130.9377 | 0.82418 | 14 | 0.84459 | 0.09853 | 62.984 | 4042080 | 14 | 10.27131 | 5938 |
| RBPJ | 41.67992 | 1 | 130.9377 | 0.82418 | 14 | 0.84459 | 0.09853 | 62.444 | 4042080 | 14 | 10.27131 | 5938 |
| MAML1 | 41.67992 | 1 | 130.9377 | 0.82418 | 14 | 0.84459 | 0.09853 | 61.573 | 4042080 | 14 | 10.27131 | 5938 |
| GALNT4 | 41.67992 | 1 | 130.9377 | 0.82418 | 14 | 0.84459 | 0.09853 | 64.873 | 4042080 | 14 | 10.27131 | 5938 |
| MANBA | 447.72383 | 3 | 137.9377 | 0.74725 | 14 | 0.86857 | 0.09853 | 52.322 | 4.79E+08 | 13 | 10.44866 | 4850 |
| SETD1B | 758.29822 | 2 | 122.14964 | 0.78022 | 14 | 0.90689 | 0.08957 | 76.54 | 4.79E+08 | 13 | 9.90677 | 25304 |
| SETD1A | 2.15329 | 1 | 116.57583 | 0.95604 | 14 | 0.97972 | 0.08957 | 74.614 | 6.23E+09 | 14 | 9.66045 | 50 |
| KMT2D | 145.53988 | 3 | 124.05437 | 0.74725 | 14 | 0.76576 | 0.09853 | 52.746 | 3644640 | 14 | 10.00776 | 20856 |
| B3GAT1 | 145.07082 | 1 | 124.1877 | 0.72527 | 14 | 0.74324 | 0.09853 | 47.843 | 3639720 | 14 | 10.02746 | 17082 |
| B3GAT2 | 145.53988 | 1 | 124.05437 | 0.74725 | 14 | 0.76576 | 0.09853 | 50.799 | 3644640 | 14 | 10.00776 | 20856 |
| LAMA1 | 849.46931 | 1 | 142.37262 | 0.54945 | 14 | 0.56306 | 0.12316 | 68.054 | 40926 | 14 | 10.59152 | 71808 |
| PPP1R12A | 4841.68702 | 14 | 148.90595 | 0.57143 | 14 | 0.58558 | 0.12316 | 73.826 | 3628826 | 14 | 10.75902 | 572668 |
| Pathways of neurodegeneration | 4841.68702 | 1 | 148.90595 | 0.57143 | 14 | 0.58558 | 0.12316 | 77.729 | 3628826 | 14 | 10.75902 | 572668 |
| B4GALT6 | 3763.91371 | 3 | 130.63056 | 0.31868 | 14 | 0.8164 | 0.09853 | 63.624 | 40326 | 8 | 10.16786 | 53392 |
| GCNT2 | 1024.27861 | 2 | 139.74325 | 0.21795 | 13 | 0.358 | 0.10947 | 52.991 | 135 | 9 | 10.52256 | 31230 |
| CEACAM8 | 680.5722 | 1 | 127.0377 | 0.60256 | 13 | 0.68784 | 0.09853 | 51.347 | 367945 | 12 | 10.11367 | 27584 |
| TMEM30A | 111.29575 | 1 | 137.27103 | 0.87179 | 13 | 0.86857 | 0.09853 | 52.915 | 4.79E+08 | 13 | 10.44373 | 1216 |
| CMTM6 | 100.99406 | 1 | 138.4377 | 0.91026 | 13 | 0.90689 | 0.09853 | 49.407 | 4.79E+08 | 13 | 10.47329 | 2718 |
| GALNT7 | 100.99406 | 1 | 138.4377 | 0.91026 | 13 | 0.90689 | 0.09853 | 54.035 | 4.79E+08 | 13 | 10.47329 | 2718 |
| CD68 | 185.55012 | 1 | 136.77103 | 0.85897 | 13 | 0.8558 | 0.09853 | 49.605 | 4.79E+08 | 13 | 10.43635 | 3296 |
| RAB3D | 100.99406 | 1 | 138.4377 | 0.91026 | 13 | 0.90689 | 0.09853 | 51.388 | 4.79E+08 | 13 | 10.47329 | 2718 |
| GALNT3 | 8218.71009 | 35 | 138.10278 | 0.75641 | 13 | 0.86346 | 0.10947 | 75.308 | 3.99E+07 | 12 | 10.51517 | 225624 |
| LAMA2 | 2.19627 | 1 | 110.55202 | 0.91026 | 13 | 0.90689 | 0.08957 | 71.431 | 4.79E+08 | 13 | 9.50528 | 252 |
| LAMA5 | 0.1 | 1 | 127.1877 | 0.98718 | 13 | 0.98353 | 0.09853 | 64.79 | 9.58E+08 | 13 | 10.15801 | 2 |
| TADA3 | 606.18965 | 5 | 141.87262 | 0.61538 | 13 | 0.61311 | 0.12316 | 64.313 | 40920 | 13 | 10.58906 | 49784 |
| GLA | 606.18965 | 1 | 141.87262 | 0.61538 | 13 | 0.61311 | 0.12316 | 65.019 | 40920 | 13 | 10.58906 | 49784 |
| Regulation of actin cytoskeleton | 3354.10346 | 5 | 150.09325 | 0.08974 | 13 | 0.47366 | 0.10947 | 51.712 | 21 | 4 | 10.74424 | 96526 |
| CDC5L | 5184.83203 | 12 | 142.14325 | 0.26923 | 13 | 0.35633 | 0.10947 | 40.524 | 166 | 11 | 10.59152 | 54294 |
| CEACAM6 | 1133.42033 | 2 | 140.87659 | 0.54545 | 12 | 0.85919 | 0.10947 | 52.376 | 362883 | 9 | 10.59399 | 27566 |
| B4GALT7 | 995.35398 | 5 | 140.70992 | 0.25758 | 12 | 0.24879 | 0.10947 | 45.254 | 50 | 12 | 10.59399 | 11868 |
| CAPZB | 3.04418 | 1 | 121.5925 | 0.90909 | 12 | 0.8781 | 0.08957 | 59.243 | 3991680 | 12 | 9.94372 | 68 |
| Notch signaling pathway | 116.8476 | 3 | 109.59369 | 0.78788 | 12 | 0.76102 | 0.08957 | 62.696 | 3628920 | 12 | 9.43385 | 26936 |
| ACTR1B | 116.8476 | 1 | 109.59369 | 0.78788 | 12 | 0.76102 | 0.08957 | 62.011 | 3628920 | 12 | 9.43385 | 26936 |
| GALNT6 | 636.20808 | 3 | 125.12103 | 0.54545 | 12 | 0.52686 | 0.09853 | 29.261 | 360 | 12 | 10.1654 | 34236 |
| GALNT11 | 636.20808 | 1 | 125.12103 | 0.54545 | 12 | 0.52686 | 0.09853 | 30.918 | 360 | 12 | 10.1654 | 34236 |
| GALNT9 | 44.74332 | 1 | 118.60437 | 0.77273 | 12 | 0.74638 | 0.09853 | 45.231 | 80784 | 12 | 9.91416 | 3614 |
| GALNT12 | 0 | 1 | 136.27103 | 1 | 12 | 0.96591 | 0.09853 | 48.037 | 4.79E+08 | 12 | 10.43388 | 0 |
| GALNT15 | 2.19627 | 1 | 107.32702 | 0.89394 | 12 | 0.86346 | 0.08957 | 71.906 | 3.99E+07 | 12 | 9.38705 | 252 |
| WBSCR17 | 2.19627 | 1 | 107.32702 | 0.89394 | 12 | 0.86346 | 0.08957 | 65.549 | 3.99E+07 | 12 | 9.38705 | 252 |
| GALNT10 | 2.19627 | 1 | 107.32702 | 0.89394 | 12 | 0.86346 | 0.08957 | 68.544 | 3.99E+07 | 12 | 9.38705 | 252 |
| GALNT5 | 2.19627 | 1 | 107.32702 | 0.89394 | 12 | 0.86346 | 0.08957 | 67.634 | 3.99E+07 | 12 | 9.38705 | 252 |
| GALNT8 | 2.19627 | 1 | 107.32702 | 0.89394 | 12 | 0.86346 | 0.08957 | 70.755 | 3.99E+07 | 12 | 9.38705 | 252 |
| GALNT18 | 2.19627 | 1 | 107.32702 | 0.89394 | 12 | 0.86346 | 0.08957 | 69.987 | 3.99E+07 | 12 | 9.38705 | 252 |
| KMT2C | 2.19627 | 1 | 107.32702 | 0.89394 | 12 | 0.86346 | 0.08957 | 71.209 | 3.99E+07 | 12 | 9.38705 | 252 |
| XYLT2 | 2.19627 | 1 | 107.32702 | 0.89394 | 12 | 0.86346 | 0.08957 | 68.31 | 3.99E+07 | 12 | 9.38705 | 252 |
| ST6GALNAC4 | 2.19627 | 1 | 107.32702 | 0.89394 | 12 | 0.86346 | 0.08957 | 69.94 | 3.99E+07 | 12 | 9.38705 | 252 |
| ST6GALNAC3 | 2.19627 | 1 | 107.32702 | 0.89394 | 12 | 0.86346 | 0.08957 | 72.989 | 3.99E+07 | 12 | 9.38705 | 252 |
| MCRS1 | 69.37202 | 1 | 125.6377 | 0.65152 | 12 | 0.6293 | 0.09853 | 44.847 | 3408 | 12 | 10.10382 | 1148 |
| B3GAT3 | 63.98792 | 1 | 121.3877 | 0.84848 | 12 | 0.81956 | 0.09853 | 46.287 | 3634560 | 12 | 9.97327 | 8358 |
| BAP1 | 4957.51429 | 7 | 77.83254 | 0.37879 | 12 | 0.54881 | 0.09853 | 3.183 | 270 | 7 | 7.72442 | 526566 |
| CD55 | 2000.53654 | 5 | 133.91548 | 0.4697 | 12 | 0.8164 | 0.12316 | 54.439 | 40327 | 8 | 10.44373 | 25986 |
| Salmonella infection | 353.91048 | 1 | 128.24881 | 0.72727 | 12 | 0.70248 | 0.12316 | 69.229 | 3628806 | 12 | 10.23437 | 5956 |
| IL2RG | 1.78656 | 1 | 131.34881 | 0.9697 | 12 | 0.93664 | 0.12316 | 70.15 | 4.35E+07 | 12 | 10.34275 | 210 |
| IL2RB | 1511.7984 | 1 | 141.81548 | 0.83333 | 12 | 0.93324 | 0.12316 | 71.793 | 3.99E+07 | 11 | 10.6063 | 171894 |
| DAG1 | 22.21827 | 1 | 119.42103 | 0.90909 | 12 | 0.8781 | 0.09853 | 43.716 | 1451520 | 12 | 10.01761 | 986 |
| DPM2 | 37.18074 | 1 | 116.86389 | 0.92424 | 12 | 0.89273 | 0.09853 | 46.903 | 4354560 | 12 | 9.90923 | 5888 |
| Rap1 signaling pathway | 791.18326 | 3 | 127.06944 | 0.60606 | 12 | 0.5854 | 0.10947 | 45.497 | 362888 | 12 | 10.24176 | 27034 |
| XYLT1 | 760.15959 | 1 | 123.28056 | 0.67273 | 11 | 0.85919 | 0.09853 | 44.612 | 362882 | 9 | 10.09889 | 12192 |
| B3GALT6 | 377.79524 | 1 | 117.9925 | 0.49091 | 11 | 0.76834 | 0.08957 | 45.744 | 5064 | 7 | 9.8846 | 8364 |
| KMT2B | 604.35782 | 1 | 124.62103 | 0.54545 | 11 | 0.50904 | 0.09853 | 28.436 | 264 | 11 | 10.16294 | 33690 |
| B3GALT2 | 34.59096 | 1 | 115.97103 | 0.83636 | 11 | 0.78053 | 0.09853 | 41.173 | 80640 | 11 | 9.81071 | 1362 |
| B3GALT1 | 34.59096 | 1 | 115.97103 | 0.83636 | 11 | 0.78053 | 0.09853 | 39.259 | 80640 | 11 | 9.81071 | 1362 |
| PHF20 | 1091.76398 | 1 | 127.75437 | 0.43636 | 11 | 0.47886 | 0.09853 | 33.713 | 169 | 10 | 10.23929 | 36556 |
| KANSL1 | 338.1539 | 2 | 138.15595 | 0.36364 | 11 | 0.33936 | 0.12316 | 54.627 | 56 | 11 | 10.5127 | 15870 |
| CSRP2BP | 92.17149 | 1 | 120.75437 | 0.78182 | 11 | 0.72963 | 0.09853 | 40.665 | 367922 | 11 | 9.98313 | 2110 |
| YEATS2 | 92.17149 | 1 | 120.75437 | 0.78182 | 11 | 0.72963 | 0.09853 | 38.067 | 367922 | 11 | 9.98313 | 2110 |
| Huntington disease | 607.83814 | 1 | 131.27103 | 0.49091 | 11 | 0.76834 | 0.09853 | 63.252 | 5064 | 7 | 10.24668 | 65912 |
| ASXL1 | 927.51935 | 4 | 114.7925 | 0.30909 | 11 | 0.57059 | 0.08957 | 46.488 | 136 | 6 | 9.72203 | 68088 |
| VAMP2 | 24.12841 | 1 | 126.47103 | 0.83636 | 11 | 0.78053 | 0.09853 | 53.72 | 82800 | 11 | 10.1654 | 9256 |
| IL2RA | 24.12841 | 1 | 126.47103 | 0.83636 | 11 | 0.78053 | 0.09853 | 57.899 | 82800 | 11 | 10.1654 | 9256 |
| Th1 and Th2 cell differentiation | 63.88678 | 1 | 125.1377 | 0.63636 | 11 | 0.59388 | 0.09853 | 41.211 | 1728 | 11 | 10.10136 | 1084 |
| ST6GAL1 | 53.51558 | 1 | 120.05437 | 0.90909 | 11 | 0.8484 | 0.09853 | 43.117 | 3629520 | 11 | 9.9585 | 7012 |
| IL2 | 651.64534 | 2 | 136.47659 | 0.56364 | 11 | 0.52601 | 0.10947 | 42.536 | 1466 | 11 | 10.47576 | 13622 |
| ALG5 | 4692.69048 | 7 | 77.16587 | 0.41818 | 11 | 0.39027 | 0.09853 | 3.166 | 180 | 11 | 7.7195 | 215480 |
| IGF2 | 0.2 | 1 | 127.74881 | 0.98182 | 11 | 0.91628 | 0.12316 | 69.072 | 7257600 | 11 | 10.2319 | 2 |
| ST8SIA1 | 0 | 1 | 130.84881 | 1 | 11 | 0.93324 | 0.12316 | 70.447 | 3.99E+07 | 11 | 10.34028 | 0 |
| DR1 | 391.60111 | 7 | 122.52659 | 0.33333 | 10 | 0.57059 | 0.10947 | 28.164 | 79 | 6 | 10.12353 | 10376 |
| EXT2 | 548.54108 | 9 | 141.52659 | 0.26667 | 10 | 0.28529 | 0.10947 | 53.874 | 36 | 6 | 10.64079 | 46248 |
| EXT1 | 527.02896 | 3 | 143.59325 | 0.48889 | 10 | 0.64146 | 0.10947 | 58.166 | 842 | 8 | 10.65064 | 25980 |
| VAMP7 | 399.5125 | 1 | 136.89325 | 0.26667 | 10 | 0.2864 | 0.10947 | 44.382 | 27 | 9 | 10.5127 | 9996 |
| FOXK2 | 604.19156 | 3 | 137.67659 | 0.24444 | 10 | 0.38896 | 0.10947 | 51.584 | 27 | 5 | 10.48068 | 6680 |
| Thyroid hormone signaling pathway | 0.85714 | 1 | 112.69964 | 0.93333 | 10 | 0.83801 | 0.08957 | 36.418 | 367920 | 10 | 9.66045 | 6 |
| ALG3 | 3025.51238 | 7 | 115.2425 | 0.33333 | 10 | 0.58344 | 0.08957 | 20.394 | 73 | 5 | 9.8181 | 51042 |
| TLN1 | 518.74339 | 1 | 125.05437 | 0.64444 | 10 | 0.69213 | 0.09853 | 40.579 | 1681 | 9 | 10.12599 | 3444 |
| Cytokine-cytokine receptor interaction | 1618.19719 | 2 | 110.71389 | 0.31111 | 10 | 0.58344 | 0.09853 | 10.72 | 61 | 5 | 9.76144 | 17868 |
| UGCG | 452.21181 | 1 | 136.45437 | 0.33333 | 10 | 0.43736 | 0.09853 | 42.035 | 82 | 8 | 10.42403 | 11578 |
| THBS2 | 411.76227 | 2 | 134.80992 | 0.66667 | 10 | 0.59858 | 0.10947 | 43.385 | 1464 | 10 | 10.45112 | 10140 |
| UGT8 | 129.33333 | 1 | 68.28297 | 0.35556 | 10 | 0.38186 | 0.08957 | 2.592 | 61 | 9 | 6.79828 | 400 |
| Breast cancer | 21518 | 30 | 127.82619 | 0.8 | 10 | 0.85919 | 0.14075 | 63.519 | 362881 | 9 | 10.33043 | 1338176 |
| FGFR2 | 2382 | 4 | 126.31905 | 0.8 | 10 | 0.85919 | 0.14075 | 62.626 | 362881 | 9 | 10.26639 | 72006 |
| F2 | 4061.08177 | 7 | 124.94325 | 0.31111 | 10 | 0.66569 | 0.10947 | 23.953 | 244 | 6 | 10.21713 | 58206 |
| MSL1 | 575.4259 | 1 | 118.48297 | 0.36111 | 9 | 0.57059 | 0.08957 | 28.712 | 75 | 6 | 9.85997 | 7092 |
| MSL2 | 2674.80015 | 4 | 130.2877 | 0.33333 | 9 | 0.34989 | 0.09853 | 33.068 | 39 | 8 | 10.29102 | 35928 |
| HS3ST3B1 | 1239.94442 | 8 | 129.06548 | 0.77778 | 9 | 0.8164 | 0.12316 | 51.728 | 40321 | 8 | 10.31319 | 6182 |
| HS3ST3A1 | 0 | 1 | 112.19964 | 1 | 9 | 0.85919 | 0.08957 | 40.505 | 362880 | 9 | 9.65799 | 0 |
| CXXC1 | 0 | 1 | 112.19964 | 1 | 9 | 0.85919 | 0.08957 | 38.173 | 362880 | 9 | 9.65799 | 0 |
| ASXL2 | 43.98124 | 1 | 119.61944 | 0.58333 | 9 | 0.5012 | 0.10947 | 28.819 | 186 | 9 | 10.01515 | 264 |
| FOXK1 | 37.74619 | 1 | 113.22583 | 0.77778 | 9 | 0.8164 | 0.08957 | 59.559 | 40321 | 8 | 9.64568 | 3000 |
| ECM-receptor interaction | 18.69671 | 1 | 121.1377 | 0.80556 | 9 | 0.69213 | 0.09853 | 35.656 | 1680 | 9 | 10.03485 | 404 |
| B4GALT5 | 16.99552 | 1 | 120.22103 | 0.80556 | 9 | 0.69213 | 0.09853 | 34.865 | 1680 | 9 | 10.00283 | 378 |
| CFB | 4.60291 | 1 | 112.72341 | 0.91667 | 9 | 0.78759 | 0.09853 | 37.162 | 15120 | 9 | 9.6777 | 52 |
| ARSA | 0 | 1 | 123.86548 | 1 | 9 | 0.85919 | 0.12316 | 64.643 | 362880 | 9 | 10.18018 | 0 |
| Human T-cell leukemia virus 1 infection | 0 | 1 | 123.86548 | 1 | 9 | 0.85919 | 0.12316 | 63.991 | 362880 | 9 | 10.18018 | 0 |
| cAMP signaling pathway | 1419.66823 | 1 | 129.01944 | 0.5 | 9 | 0.4296 | 0.10947 | 31.118 | 156 | 9 | 10.3058 | 17040 |
| KANSL2 | 153.48703 | 1 | 126.75437 | 0.28571 | 8 | 0.33284 | 0.09853 | 42.064 | 16 | 6 | 10.15308 | 4028 |
| KANSL3 | 235.76568 | 1 | 129.75992 | 0.21429 | 8 | 0.2842 | 0.10947 | 34.225 | 13 | 4 | 10.3255 | 3396 |
| GALNT14 | 53.23246 | 1 | 120.8377 | 0.5 | 8 | 0.4082 | 0.09853 | 35.353 | 128 | 8 | 9.99544 | 1862 |
| HS6ST2 | 627.86194 | 1 | 129.2377 | 0.28571 | 8 | 0.56839 | 0.09853 | 34.28 | 29 | 4 | 10.27624 | 13752 |
| HS3ST5 | 2.60404 | 1 | 111.50675 | 0.89286 | 8 | 0.72893 | 0.09853 | 23.789 | 5160 | 8 | 9.70972 | 20 |
| HS6ST3 | 2.60404 | 1 | 111.50675 | 0.89286 | 8 | 0.72893 | 0.09853 | 25.372 | 5160 | 8 | 9.70972 | 20 |
| HS2ST1 | 25.60054 | 1 | 124.25992 | 0.78571 | 8 | 0.64146 | 0.10947 | 32.906 | 480 | 8 | 10.19988 | 1156 |
| HS3ST4 | 50.57226 | 1 | 117.10437 | 0.75 | 8 | 0.6123 | 0.09853 | 21.754 | 192 | 8 | 9.97327 | 4978 |
| NDST2 | 0 | 1 | 108.78297 | 1 | 8 | 0.8164 | 0.08957 | 32.286 | 40320 | 8 | 9.53483 | 0 |
| NDST3 | 0 | 1 | 108.78297 | 1 | 8 | 0.8164 | 0.08957 | 35.079 | 40320 | 8 | 9.53483 | 0 |
| NDST4 | 601.26401 | 1 | 108.80083 | 0.75 | 8 | 0.76834 | 0.08957 | 50.511 | 5041 | 7 | 9.50774 | 18844 |
| NDST1 | 230.02776 | 1 | 114.26944 | 0.75 | 8 | 0.6123 | 0.10947 | 15.517 | 768 | 8 | 9.92647 | 8014 |
| HS3ST2 | 230.02776 | 1 | 114.26944 | 0.75 | 8 | 0.6123 | 0.10947 | 13.418 | 768 | 8 | 9.92647 | 8014 |
| HS6ST1 | 230.02776 | 1 | 114.26944 | 0.75 | 8 | 0.6123 | 0.10947 | 16.339 | 768 | 8 | 9.92647 | 8014 |
| HS3ST1 | 0 | 1 | 105.32702 | 1 | 8 | 0.8164 | 0.08957 | 58.516 | 40320 | 8 | 9.37719 | 0 |
| MBD6 | 113.95474 | 1 | 127.44325 | 0.64286 | 8 | 0.52483 | 0.10947 | 26.23 | 252 | 8 | 10.23683 | 1566 |
| PPP1R2 | 113.95474 | 1 | 127.44325 | 0.64286 | 8 | 0.52483 | 0.10947 | 27.545 | 252 | 8 | 10.23683 | 1566 |
| PPP1R11 | 31.13267 | 1 | 112.55916 | 0.75 | 8 | 0.76834 | 0.08957 | 55.015 | 5041 | 7 | 9.64075 | 2646 |
| PPP1R12B | 2.14564 | 1 | 119.80437 | 0.92857 | 8 | 0.75809 | 0.09853 | 32.547 | 2880 | 8 | 10.02007 | 30 |
| CHST4 | 0 | 1 | 123.36548 | 1 | 8 | 0.8164 | 0.12316 | 60.763 | 40320 | 8 | 10.17771 | 0 |
| B3GNT7 | 0 | 1 | 123.36548 | 1 | 8 | 0.8164 | 0.12316 | 56.67 | 40320 | 8 | 10.17771 | 0 |
| CHST12 | 0 | 1 | 123.36548 | 1 | 8 | 0.8164 | 0.12316 | 58.068 | 40320 | 8 | 10.17771 | 0 |
| CHST13 | 0 | 1 | 123.36548 | 1 | 8 | 0.8164 | 0.12316 | 62.368 | 40320 | 8 | 10.17771 | 0 |
| CHST11 | 0 | 1 | 123.36548 | 1 | 8 | 0.8164 | 0.12316 | 60.031 | 40320 | 8 | 10.17771 | 0 |
| IL15 | 0 | 1 | 123.36548 | 1 | 8 | 0.8164 | 0.12316 | 60.735 | 40320 | 8 | 10.17771 | 0 |
| BMPR1A | 0 | 1 | 123.36548 | 1 | 8 | 0.8164 | 0.12316 | 60.818 | 40320 | 8 | 10.17771 | 0 |
| BMPR1B | 0 | 1 | 123.36548 | 1 | 8 | 0.8164 | 0.12316 | 57.057 | 40320 | 8 | 10.17771 | 0 |
| Vascular smooth muscle contraction | 0 | 1 | 123.36548 | 1 | 8 | 0.8164 | 0.12316 | 60.086 | 40320 | 8 | 10.17771 | 0 |
| B3GALNT1 | 0 | 1 | 123.36548 | 1 | 8 | 0.8164 | 0.12316 | 59.887 | 40320 | 8 | 10.17771 | 0 |
| Platelet activation | 0 | 1 | 123.36548 | 1 | 8 | 0.8164 | 0.12316 | 57.173 | 40320 | 8 | 10.17771 | 0 |
| Alcoholism | 0 | 1 | 123.36548 | 1 | 8 | 0.8164 | 0.12316 | 60.719 | 40320 | 8 | 10.17771 | 0 |
| Signaling pathways regulating pluripotency of stem cells | 977.45207 | 1 | 129.15437 | 0.5 | 8 | 0.51223 | 0.09853 | 38.574 | 99 | 7 | 10.29595 | 20820 |
| PPP1R15B | 164.72283 | 1 | 115.9377 | 0.57143 | 7 | 0.57059 | 0.09853 | 22.519 | 73 | 6 | 9.94372 | 4194 |
| ST6GALNAC2 | 553.97787 | 1 | 128.65437 | 0.52381 | 7 | 0.64826 | 0.09853 | 35.245 | 122 | 5 | 10.26393 | 5740 |
| TADA2A | 188.65432 | 2 | 128.09325 | 0.47619 | 7 | 0.36588 | 0.10947 | 27.551 | 32 | 7 | 10.36738 | 2632 |
| B3GNT8 | 119.05207 | 1 | 129.55992 | 0.47619 | 7 | 0.64826 | 0.10947 | 42.769 | 122 | 5 | 10.36738 | 1774 |
| ABO | 39.2223 | 1 | 128.62659 | 0.28571 | 7 | 0.28529 | 0.10947 | 39.893 | 13 | 6 | 10.31811 | 1044 |
| A4GNT | 356.3907 | 1 | 124.74325 | 0.47619 | 7 | 0.64826 | 0.10947 | 29.355 | 122 | 5 | 10.19249 | 3890 |
| UST | 196.60256 | 1 | 129.32103 | 0.47619 | 7 | 0.36588 | 0.09853 | 32.732 | 32 | 7 | 10.28856 | 5646 |
| CSGALNACT2 | 684.95685 | 5 | 106.64722 | 0.38095 | 7 | 0.47366 | 0.09853 | 9.881 | 18 | 4 | 9.66292 | 9386 |
| CSGALNACT1 | 227.7229 | 1 | 118.87103 | 0.52381 | 7 | 0.64826 | 0.09853 | 29.224 | 122 | 5 | 10.03978 | 5798 |
| CFI | 0 | 1 | 111.00675 | 1 | 7 | 0.76834 | 0.09853 | 23.022 | 5040 | 7 | 9.70725 | 0 |
| DPM1 | 0 | 1 | 104.66035 | 1 | 7 | 0.76834 | 0.08957 | 54.385 | 5040 | 7 | 9.37227 | 0 |
| DPM3 | 50.28655 | 2 | 116.60437 | 0.71429 | 7 | 0.54881 | 0.09853 | 18.341 | 96 | 7 | 9.97081 | 4976 |
| IL15RA | 0 | 1 | 111.19964 | 1 | 7 | 0.76834 | 0.08957 | 32.694 | 5040 | 7 | 9.65307 | 0 |
| ST8SIA5 | 188.40301 | 2 | 113.76944 | 0.85714 | 7 | 0.65858 | 0.10947 | 15.548 | 744 | 7 | 9.92401 | 6934 |
| JAK-STAT signaling pathway | 3.08333 | 1 | 116.78611 | 0.7619 | 7 | 0.5854 | 0.10947 | 21.272 | 168 | 7 | 9.98066 | 14 |
| Cushing syndrome | 131.45626 | 1 | 123.92659 | 0.42857 | 7 | 0.32929 | 0.10947 | 34.083 | 20 | 7 | 10.18018 | 1418 |
| Vasopressin-regulated water reabsorption | 747.77604 | 3 | 134.44325 | 0.42857 | 7 | 0.56839 | 0.10947 | 36.402 | 30 | 4 | 10.47822 | 23210 |
| DPAGT1 | 1073.52726 | 3 | 127.87659 | 0.42857 | 7 | 0.56839 | 0.10947 | 31.595 | 30 | 4 | 10.33043 | 30724 |
| Amoebiasis | 937.96688 | 1 | 133.85992 | 0.42857 | 7 | 0.56839 | 0.10947 | 38.262 | 30 | 4 | 10.46837 | 29418 |
| TGF-beta signaling pathway | 22.04679 | 2 | 98.90931 | 0.61905 | 7 | 0.47564 | 0.0821 | 31.077 | 128 | 7 | 9.07915 | 1270 |
| Lysosome | 0 | 1 | 104.66035 | 1 | 7 | 0.76834 | 0.08957 | 52.508 | 5040 | 7 | 9.37227 | 0 |
| Long-term potentiation | 2.66667 | 1 | 107.80278 | 0.80952 | 7 | 0.62199 | 0.10947 | 19.091 | 726 | 7 | 9.66538 | 8 |
| MGAT5B | 2.66667 | 1 | 107.80278 | 0.80952 | 7 | 0.62199 | 0.10947 | 19.593 | 726 | 7 | 9.66538 | 8 |
| POMGNT1 | 13517.42857 | 24 | 86.43016 | 0.52381 | 7 | 0.64826 | 0.10947 | 3.203 | 122 | 5 | 8.62101 | 812668 |
| FUT8 | 229.51429 | 2 | 75.16587 | 0.71429 | 7 | 0.54881 | 0.09853 | 2.357 | 150 | 7 | 7.70964 | 47886 |
| CFD | 229.51429 | 1 | 75.16587 | 0.71429 | 7 | 0.54881 | 0.09853 | 2.428 | 150 | 7 | 7.70964 | 47886 |
| Sphingolipid metabolism | 0 | 1 | 99.0425 | 1 | 7 | 0.76834 | 0.08957 | 39.069 | 5040 | 7 | 9.24172 | 0 |
| Prostate cancer | 0 | 1 | 104.66035 | 1 | 7 | 0.76834 | 0.08957 | 50.979 | 5040 | 7 | 9.37227 | 0 |
| Lysine degradation | 86.0472 | 1 | 128.60992 | 0.33333 | 6 | 0.46346 | 0.10947 | 32.902 | 10 | 3 | 10.35752 | 3316 |
| SNARE interactions in vesicular transport | 80.87372 | 1 | 121.20992 | 0.6 | 6 | 0.42794 | 0.10947 | 26.625 | 32 | 6 | 10.13092 | 2010 |
| STX17 | 30.07221 | 1 | 130.20992 | 0.4 | 6 | 0.28529 | 0.10947 | 36.837 | 12 | 6 | 10.40186 | 3420 |
| STX16 | 0 | 1 | 103.26627 | 1 | 6 | 0.71324 | 0.09853 | 21.939 | 720 | 6 | 9.48557 | 0 |
| MFNG | 0 | 1 | 112.85437 | 1 | 6 | 0.71324 | 0.09853 | 22.306 | 720 | 6 | 9.90184 | 0 |
| RFNG | 0 | 1 | 112.85437 | 1 | 6 | 0.71324 | 0.09853 | 22.93 | 720 | 6 | 9.90184 | 0 |
| LFNG | 0 | 1 | 112.85437 | 1 | 6 | 0.71324 | 0.09853 | 20.189 | 720 | 6 | 9.90184 | 0 |
| CHST3 | 0 | 1 | 112.82583 | 1 | 6 | 0.71324 | 0.08957 | 33.427 | 720 | 6 | 9.8181 | 0 |
| CHST7 | 0 | 1 | 112.82583 | 1 | 6 | 0.71324 | 0.08957 | 30.515 | 720 | 6 | 9.8181 | 0 |
| CHSY1 | 0 | 1 | 112.82583 | 1 | 6 | 0.71324 | 0.08957 | 31.466 | 720 | 6 | 9.8181 | 0 |
| CHSY3 | 35.47844 | 1 | 110.50675 | 0.6 | 6 | 0.42794 | 0.09853 | 19.729 | 30 | 6 | 9.70479 | 370 |
| CHPF2 | 7.34286 | 1 | 118.33611 | 0.6 | 6 | 0.42794 | 0.10947 | 20.005 | 30 | 6 | 10.09643 | 24 |
| CHPF | 158 | 2 | 64.56631 | 0.93333 | 6 | 0.66569 | 0.08957 | 1.954 | 240 | 6 | 6.75641 | 47870 |
| PIGC | 158 | 1 | 64.56631 | 0.93333 | 6 | 0.66569 | 0.08957 | 1.944 | 240 | 6 | 6.75641 | 47870 |
| PIGP | 158 | 1 | 64.56631 | 0.93333 | 6 | 0.66569 | 0.08957 | 1.984 | 240 | 6 | 6.75641 | 47870 |
| PIGA | 158 | 1 | 64.56631 | 0.93333 | 6 | 0.66569 | 0.08957 | 1.946 | 240 | 6 | 6.75641 | 47870 |
| PIGL | 158 | 1 | 64.56631 | 0.93333 | 6 | 0.66569 | 0.08957 | 1.953 | 240 | 6 | 6.75641 | 47870 |
| PIGH | 0 | 1 | 109.16944 | 1 | 6 | 0.71324 | 0.10947 | 12.846 | 720 | 6 | 9.76391 | 0 |
| RGMB | 1325.64961 | 5 | 112.24722 | 0.4 | 6 | 0.56839 | 0.09853 | 12.288 | 26 | 4 | 9.82302 | 30726 |
| RGMA | 0 | 1 | 109.16944 | 1 | 6 | 0.71324 | 0.10947 | 11.289 | 720 | 6 | 9.76391 | 0 |
| ADAMTSL1 | 0 | 1 | 107.30278 | 1 | 6 | 0.71324 | 0.10947 | 12.201 | 720 | 6 | 9.66292 | 0 |
| ADAMTS2 | 0 | 1 | 107.30278 | 1 | 6 | 0.71324 | 0.10947 | 11.959 | 720 | 6 | 9.66292 | 0 |
| CFP | 0 | 1 | 107.30278 | 1 | 6 | 0.71324 | 0.10947 | 11.867 | 720 | 6 | 9.66292 | 0 |
| ADAMTS18 | 0 | 1 | 107.30278 | 1 | 6 | 0.71324 | 0.10947 | 13.69 | 720 | 6 | 9.66292 | 0 |
| GALNS | 10.19802 | 1 | 95.61035 | 0.8 | 6 | 0.57059 | 0.08957 | 5.64 | 60 | 6 | 9.18999 | 256 |
| CERS4 | 10.19802 | 1 | 95.61035 | 0.8 | 6 | 0.57059 | 0.08957 | 5.181 | 60 | 6 | 9.18999 | 256 |
| SMPD3 | 3.08333 | 1 | 116.28611 | 0.73333 | 6 | 0.52304 | 0.10947 | 20.405 | 54 | 6 | 9.9782 | 12 |
| CFH | 20.24516 | 1 | 123.89325 | 0.93333 | 6 | 0.66569 | 0.10947 | 24.455 | 240 | 6 | 10.17525 | 228 |
| DOLK | 20.24516 | 1 | 123.89325 | 0.93333 | 6 | 0.66569 | 0.10947 | 25.439 | 240 | 6 | 10.17525 | 228 |
| POFUT2 | 1592 | 3 | 122.30437 | 0.46667 | 6 | 0.56839 | 0.09853 | 31.138 | 26 | 4 | 10.11121 | 44924 |
| A4GALT | 35.37619 | 1 | 106.48056 | 0.46667 | 6 | 0.45378 | 0.09853 | 10.03 | 19 | 5 | 9.66538 | 338 |
| Staphylococcus aureus infection | 3867.5 | 6 | 74.91587 | 0.66667 | 6 | 0.64826 | 0.09853 | 2.444 | 121 | 5 | 7.71211 | 167636 |
| SSU72 | 333.83333 | 1 | 65.61631 | 0.46667 | 6 | 0.45378 | 0.08957 | 2.018 | 27 | 5 | 6.77857 | 24164 |
| MGAT4B | 5213.57143 | 3 | 84.88016 | 0.26667 | 6 | 0.46346 | 0.10947 | 2.664 | 9 | 3 | 8.59638 | 430326 |
| ALG10B | 372.89103 | 4 | 101.38056 | 0.66667 | 6 | 0.64826 | 0.09853 | 6.909 | 121 | 5 | 9.51759 | 22566 |
| B3GNT1 | 0.8 | 1 | 95.53294 | 0.93333 | 6 | 0.66569 | 0.09853 | 4.982 | 240 | 6 | 9.24911 | 4 |
| NEU2 | 0.8 | 1 | 95.53294 | 0.93333 | 6 | 0.66569 | 0.09853 | 4.986 | 240 | 6 | 9.24911 | 4 |
| CHST2 | 0.8 | 1 | 95.53294 | 0.93333 | 6 | 0.66569 | 0.09853 | 4.597 | 240 | 6 | 9.24911 | 4 |
| Melanoma | 0.8 | 1 | 95.53294 | 0.93333 | 6 | 0.66569 | 0.09853 | 4.599 | 240 | 6 | 9.24911 | 4 |
| CDK11A | 751.6545 | 2 | 125.15437 | 0.4 | 6 | 0.46346 | 0.09853 | 34.686 | 12 | 3 | 10.19742 | 12222 |
| Parathyroid hormone synthesis, secretion and action | 15.94407 | 1 | 126.13611 | 0.86667 | 6 | 0.61814 | 0.10947 | 33.606 | 144 | 6 | 10.27131 | 124 |
| ALG1 | 135.36759 | 1 | 125.3877 | 0.6 | 6 | 0.58344 | 0.09853 | 35.17 | 49 | 5 | 10.20974 | 3634 |
| Glycosylphosphatidylinositol (GPI)-anchor biosynthesis | 46.02058 | 1 | 121.42103 | 0.4 | 5 | 0.46346 | 0.09853 | 29.156 | 8 | 3 | 10.08165 | 1224 |
| ST6GALNAC5 | 80.10161 | 1 | 119.57103 | 0.4 | 5 | 0.46346 | 0.09853 | 25.279 | 8 | 3 | 10.02746 | 2098 |
| PPP1R7 | 3169.5 | 5 | 64.69964 | 0.3 | 5 | 0.46346 | 0.08957 | 1.704 | 8 | 3 | 6.76872 | 143872 |
| CD46 | 0 | 1 | 55.67554 | 1 | 5 | 0.64826 | 0.0821 | 1.773 | 120 | 5 | 5.78346 | 0 |
| CR1 | 0 | 1 | 91.84264 | 1 | 5 | 0.64826 | 0.0821 | 25.768 | 120 | 5 | 8.7688 | 0 |
| B3GALTL | 79.19843 | 2 | 123.75437 | 0.6 | 5 | 0.38896 | 0.09853 | 20.553 | 14 | 5 | 10.19003 | 1664 |
| SGPP1 | 0 | 1 | 109.09008 | 1 | 5 | 0.64826 | 0.09853 | 20.197 | 120 | 5 | 9.68755 | 0 |
| CERK | 695.88314 | 5 | 113.9925 | 0.6 | 5 | 0.56839 | 0.08957 | 27.916 | 25 | 4 | 9.85012 | 30188 |
| ALG12 | 22.35233 | 1 | 108.83611 | 0.7 | 5 | 0.45378 | 0.10947 | 10.759 | 26 | 5 | 9.76391 | 1812 |
| CHST15 | 9.79802 | 1 | 95.11035 | 0.8 | 5 | 0.51861 | 0.08957 | 5.656 | 30 | 5 | 9.18753 | 254 |
| ALG10 | 9.79802 | 1 | 95.11035 | 0.8 | 5 | 0.51861 | 0.08957 | 5.327 | 30 | 5 | 9.18753 | 254 |
| POFUT1 | 0 | 1 | 115.78611 | 1 | 5 | 0.64826 | 0.10947 | 20.207 | 120 | 5 | 9.97574 | 0 |
| RAPGEF4 | 0 | 1 | 115.78611 | 1 | 5 | 0.64826 | 0.10947 | 18.67 | 120 | 5 | 9.97574 | 0 |
| Chronic myeloid leukemia | 201.17944 | 1 | 102.46035 | 0.3 | 5 | 0.2842 | 0.08957 | 15.165 | 7 | 4 | 9.37966 | 3000 |
| Amphetamine addiction | 3.83333 | 1 | 64.53297 | 0.8 | 5 | 0.51861 | 0.08957 | 1.872 | 30 | 5 | 6.76626 | 18 |
| N-Glycan biosynthesis | 260.83333 | 1 | 64.53297 | 0.7 | 5 | 0.45378 | 0.08957 | 1.935 | 26 | 5 | 6.76626 | 23980 |
| B4GALNT1 | 1073.06932 | 3 | 123.18214 | 0 | 5 | 0 | 0.12316 | 22.252 | 5 | 1 | 10.22944 | 17364 |
| NCAM1 | 0 | 1 | 94.86627 | 1 | 5 | 0.64826 | 0.09853 | 4.851 | 120 | 5 | 9.24418 | 0 |
| MGAT3 | 264.66667 | 2 | 55.52554 | 0.83333 | 4 | 0.47366 | 0.0821 | 1.453 | 12 | 4 | 5.79578 | 23980 |
| MGAT2 | 264.66667 | 1 | 55.52554 | 0.83333 | 4 | 0.47366 | 0.0821 | 1.505 | 12 | 4 | 5.79578 | 23980 |
| ST6GAL2 | 264.66667 | 1 | 55.52554 | 0.83333 | 4 | 0.47366 | 0.0821 | 1.514 | 12 | 4 | 5.79578 | 23980 |
| MAN1C1 | 0 | 1 | 117.00278 | 1 | 4 | 0.56839 | 0.10947 | 15.685 | 24 | 4 | 10.08412 | 0 |
| MAN1A2 | 0 | 1 | 117.00278 | 1 | 4 | 0.56839 | 0.10947 | 16.053 | 24 | 4 | 10.08412 | 0 |
| MAN1A1 | 22.02927 | 1 | 115.97659 | 0 | 4 | 0 | 0.10947 | 20.095 | 4 | 1 | 9.98066 | 308 |
| ENSG00000086848 | 231.9424 | 1 | 114.75437 | 0.5 | 4 | 0.46346 | 0.09853 | 12.382 | 7 | 3 | 9.94864 | 8124 |
| INTS1 | 0 | 1 | 120.85437 | 1 | 4 | 0.56839 | 0.09853 | 30.094 | 24 | 4 | 10.10136 | 0 |
| ST8SIA2 | 445.66171 | 1 | 122.99325 | 0.5 | 4 | 0.46346 | 0.10947 | 27.532 | 7 | 3 | 10.2319 | 14896 |
| ALG6 | 261.83333 | 2 | 63.56631 | 0.5 | 4 | 0.46346 | 0.08957 | 1.78 | 7 | 3 | 6.75148 | 23980 |
| ALG13 | 0.66667 | 1 | 63.56631 | 0.83333 | 4 | 0.47366 | 0.08957 | 1.791 | 12 | 4 | 6.75148 | 2 |
| INHBA | 20832.8 | 29 | 100.1131 | 0.33333 | 4 | 0.30898 | 0.12316 | 8.208 | 5 | 3 | 9.48557 | 1314394 |
| MGAT5 | 157.59527 | 2 | 123.14325 | 0 | 3 | 0 | 0.10947 | 14.938 | 3 | 1 | 10.26146 | 2174 |
| MGAT1 | 0 | 1 | 116.83611 | 1 | 3 | 0.46346 | 0.10947 | 15.926 | 6 | 3 | 10.08904 | 0 |
| INTS6 | 0 | 1 | 48.52703 | 1 | 3 | 0.46346 | 0.07579 | 1.391 | 6 | 3 | 4.81791 | 0 |
| GAL3ST1 | 0 | 1 | 102.53294 | 1 | 3 | 0.46346 | 0.09853 | 7.472 | 6 | 3 | 9.50035 | 0 |
| FUT7 | 0 | 1 | 104.81389 | 1 | 3 | 0.46346 | 0.09853 | 7.126 | 6 | 3 | 9.65553 | 0 |
| PROCR | 0 | 1 | 103.95916 | 1 | 3 | 0.46346 | 0.08957 | 16.078 | 6 | 3 | 9.52498 | 0 |
| POMT1 | 0 | 1 | 115.94325 | 1 | 3 | 0.46346 | 0.10947 | 19.511 | 6 | 3 | 9.99791 | 0 |
| POMT2 | 1594 | 3 | 94.78571 | 0 | 3 | 0 | 0.12316 | 7.158 | 3 | 1 | 9.29345 | 48010 |
| CFHR1 | 0 | 1 | 106.81944 | 1 | 3 | 0.46346 | 0.10947 | 8.95 | 6 | 3 | 9.79593 | 0 |
| ST3GAL5 | 0 | 1 | 106.81944 | 1 | 3 | 0.46346 | 0.10947 | 8.106 | 6 | 3 | 9.79593 | 0 |
| ALG14 | 798 | 2 | 105.96944 | 0.33333 | 3 | 0.30779 | 0.10947 | 8.114 | 3 | 2 | 9.65799 | 20246 |
| CHST14 | 0 | 1 | 114.78611 | 1 | 3 | 0.46346 | 0.10947 | 18.355 | 6 | 3 | 9.97081 | 0 |
| B3GALT4 | 32.02206 | 1 | 100.96035 | 0.66667 | 3 | 0.30898 | 0.08957 | 11.915 | 4 | 3 | 9.36734 | 330 |
| ALG8 | 5.16573 | 1 | 92.84264 | 0.33333 | 3 | 0.30779 | 0.0821 | 16.433 | 3 | 2 | 8.85993 | 44 |
| Other glycan degradation | 3.83333 | 1 | 55.57554 | 0.33333 | 3 | 0.30779 | 0.0821 | 1.488 | 3 | 2 | 5.80563 | 18 |
| EXTL3 | 0.8 | 1 | 82.13016 | 0.66667 | 3 | 0.30898 | 0.10947 | 1.827 | 4 | 3 | 8.56928 | 4 |
| RAP1GAP | 0 | 1 | 113.70437 | 1 | 2 | 0.30779 | 0.09853 | 12.526 | 2 | 2 | 9.98559 | 0 |
| RAP1GAP2 | 0 | 1 | 108.36631 | 1 | 2 | 0.30779 | 0.08957 | 7.804 | 2 | 2 | 9.73681 | 0 |
| RAB35 | 0 | 1 | 109.2877 | 1 | 2 | 0.30779 | 0.09853 | 11.936 | 2 | 2 | 9.84273 | 0 |
| KRIT1 | 0 | 1 | 113.70437 | 1 | 2 | 0.30779 | 0.09853 | 8.012 | 2 | 2 | 9.98559 | 0 |
| KL | 0 | 1 | 118.34325 | 1 | 2 | 0.30779 | 0.10947 | 15.226 | 2 | 2 | 10.13338 | 0 |
| CR2 | 1592 | 3 | 96.68297 | 0 | 2 | 0 | 0.08957 | 3.741 | 2 | 1 | 9.31808 | 19112 |
| LALBA | 798 | 2 | 77.65097 | 0 | 2 | 0 | 0.0821 | 1.385 | 2 | 1 | 8.34021 | 9558 |
| GYLTL1B | 0 | 1 | 114.28611 | 1 | 2 | 0.30779 | 0.10947 | 16.449 | 2 | 2 | 9.96835 | 0 |
| LARGE | 0 | 1 | 116.60437 | 1 | 2 | 0.30779 | 0.09853 | 18.081 | 2 | 2 | 10.02007 | 0 |
| CHST10 | 0 | 1 | 92.02583 | 1 | 2 | 0.30779 | 0.08957 | 4.029 | 2 | 2 | 9.13088 | 0 |
| GBGT1 | 0 | 1 | 92.02583 | 1 | 2 | 0.30779 | 0.08957 | 5.019 | 2 | 2 | 9.13088 | 0 |
| LHB | 0 | 1 | 106.05278 | 1 | 2 | 0.30779 | 0.10947 | 15.16 | 2 | 2 | 9.77869 | 0 |
| CHST8 | 0 | 1 | 99.7925 | 1 | 2 | 0.30779 | 0.08957 | 10.512 | 2 | 2 | 9.42646 | 0 |
| ALG11 | 17 | 1 | 62.39964 | 0 | 2 | 0 | 0.08957 | 1.371 | 2 | 1 | 6.74409 | 34 |
| ALG2 | 750 | 2 | 69.59921 | 0 | 2 | 0 | 0.09853 | 1.512 | 2 | 1 | 7.63575 | 23904 |
| UGGT2 | 0 | 1 | 1 | 0 | 1 | 0 | 0.00491 | 1.093 | 1 | 1 | 0.01474 | 0 |
| UGGT1 | 0 | 1 | 1 | 0 | 1 | 0 | 0.00491 | 1.093 | 1 | 1 | 0.01474 | 0 |
| ST8SIA3 | 0 | 1 | 110.55437 | 0 | 1 | 0 | 0.09853 | 7.438 | 1 | 1 | 9.87721 | 0 |
| ST8SIA4 | 0 | 1 | 92.32063 | 0 | 1 | 0 | 0.10947 | 3.149 | 1 | 1 | 9.24665 | 0 |
| MGEA5 | 0 | 1 | 98.11631 | 0 | 1 | 0 | 0.08957 | 5.639 | 1 | 1 | 9.3501 | 0 |
| FKTN | 0 | 1 | 1 | 0 | 1 | 0 | 0.00491 | 1.089 | 1 | 1 | 0.01474 | 0 |
| FKRP | 0 | 1 | 1 | 0 | 1 | 0 | 0.00491 | 1.089 | 1 | 1 | 0.01474 | 0 |
| EXTL2 | 0 | 1 | 75.64008 | 0 | 1 | 0 | 0.10947 | 1.69 | 1 | 1 | 8.31065 | 0 |
| EXTL1 | 0 | 1 | 75.64008 | 0 | 1 | 0 | 0.10947 | 2.033 | 1 | 1 | 8.31065 | 0 |
| CHST9 | 0 | 1 | 64.57382 | 0 | 1 | 0 | 0.07579 | 1.095 | 1 | 1 | 7.35741 | 0 |
| CHST6 | 0 | 1 | 98.25916 | 0 | 1 | 0 | 0.08957 | 5.945 | 1 | 1 | 9.34764 | 0 |
| CHST5 | 0 | 1 | 98.25916 | 0 | 1 | 0 | 0.08957 | 7.805 | 1 | 1 | 9.34764 | 0 |
| FUT11 | 0 | 1 | 82.29603 | 0 | 1 | 0 | 0.09853 | 1.639 | 1 | 1 | 8.6752 | 0 |
| CHST1 | 0 | 1 | 98.25916 | 0 | 1 | 0 | 0.08957 | 7.144 | 1 | 1 | 9.34764 | 0 |
| CHPT1 | 0 | 1 | 84.51273 | 0 | 1 | 0 | 0.08957 | 1.965 | 1 | 1 | 8.77865 | 0 |
| B4GALNT4 | 0 | 1 | 1 | 0 | 1 | 0 | 0.00491 | 1.094 | 1 | 1 | 0.01474 | 0 |
| B4GALNT3 | 0 | 1 | 1 | 0 | 1 | 0 | 0.00491 | 1.094 | 1 | 1 | 0.01474 | 0 |
| B3GALNT2 | 0 | 1 | 97.62778 | 0 | 1 | 0 | 0.10947 | 6.666 | 1 | 1 | 9.46094 | 0 |

Abbreviation: DMNC, Density of Maximum Neighborhood Component; EPC, Edge Percolated component; MCC, Maximal Clique Centrality; MNC, Maximum neighborhood component.
